# The 20th Anniversary of Pegaptanib (MacugenTM), the First Approved Aptamer Medicine: History, Recent Advances and Future Prospects of Aptamers in Therapy

**DOI:** 10.3390/pharmaceutics17030394

**Published:** 2025-03-20

**Authors:** Miklós Bege, Rasha Ghanem Kattoub, Anikó Borbás

**Affiliations:** 1 Department of Pharmaceutical Chemistry, University of Debrecen, Egyetem tér 1, 4032 Debrecen, Hungary; bege.miklos@pharm.unideb.hu (M.B.); rasha.ghanemkattoub@pharm.unideb.hu (R.G.K.); 2 Doctoral School of Pharmaceutical Sciences, Faculty of Pharmacy, University of Debrecen, Nagyerdei krt. 98., 4032 Debrecen, Hungary

**Keywords:** SELEX, aptamer, pegaptanib, vascular endothelial growth factor, avacincaptad pegol, chemical antibodies

## Abstract

In addition to classic small-molecule drugs and modern protein-based biologics, an intriguing class of medicines is the therapeutic oligonucleotides. Most approved drugs in this category are antisense oligomers or those acting via RNA interference, both of which use base hybridization. Aptamers, also known as chemical antibodies form a smaller, yet equally interesting group of oligonucleotides that can recognize a wide range of molecular targets. Despite their high potential, only two aptamers have been approved to date, pegaptanib (MacugenTM) and avacincaptad pegol (IzervayTM), both for the treatment of age-related macular degeneration (AMD). Targeting vascular endothelial growth factor (VEGF), which plays an important role in the pathogenesis of many eye diseases, pegaptanib emerged as the first anti-VEGF agent and was used in various indications, further inspiring the development of other anti-VEGF therapies. In this review, we summarize the history of the first approved aptamer medicine, pegaptanib. We describe its chemistry and track its development from the earliest stages to the preclinical phase, clinical trials, and eventual regulatory approval. Additionally, we evaluate its position among other therapeutic agents and provide a comprehensive overview of pegaptanib’s efficacy, safety, and cost-effectiveness, comparing these aspects with those of monoclonal antibodies with similar indications, bevacizumab and ranibizumab.

## 1. Introduction

Aptamers are short (10–30 nucleotide long), single-stranded (ss) oligonucleotides (either DNA or RNA) that bind to specific molecular targets. The name comes from the Latin word *aptus* (to fit) and the Greek *meros* (part or place) [1,2,3,4,5]. The development of ligand-binding nucleic acids began after the observation that short viral RNAs can target viral or host–cell proteins and modulate their activity [3,6]. The standard method for developing aptamer RNAs is the SELEX (systematic evolution of ligands by exponential enrichment). It starts from a wide range of randomly synthesized oligomers (approximately 1015 different sequences). This mixture is incubated with the target molecule, usually a protein, and then the free oligomers are separated from the bound ones, e.g., by washing. The selected sequences are amplified using reverse-transcriptase–polymerase chain reaction (RT-PCR), followed by transcription. This process is then repeated multiple times, usually 8–12 times, providing the oligomers with the most advantageous binding properties [2,3,6,7,8]. Following this, the selected aptamers can undergo further synthetic modification. The production of aptamers by the conventional SELEX is a labor-intensive and time-consuming process associated with substantial reagent costs and a low success rate. Thus, recent innovative techniques have emerged to optimize the efficiency of aptamers selection and enhance their binding affinity using fewer resources [9,10]. Methodologies based on SELEX selection include high-fidelity SELEX (Hi-Fi SELEX) [11], SELEX assisted by graphene oxide (GO-SELEX) [12], capillary electrophoresis-SELEX (CE-SELEX) [13,14], capture-SELEX [15,16], SELEX combined with fluorescent labels and magnetic beads (Flumag-SELEX) [17], inertial microfluidic SELEX (I-SELEX) [18], nuclease-assisted SELEX (NA-SELEX) [19], staggered target SELEX (ST-SELEX) [20], and SELEX using complete living cells (Cell-SELEX) [21,22]. Unlike antisense oligonucleotides, which interact with complementary nucleic acid sequences due to base-pairing, aptamers fold into secondary and tertiary structures and bind to the target based on shape complementarity and secondary interactions. This allows aptamers to target a broad range of molecules, not only limited to nucleic acids. Thus far, aptamers have been developed against a plethora of different targets, including proteins, viruses, metal ions, and small molecules, even non-metabolites such as 2,4,6-trinitrotoluene (TNT) [23,24,25,26]. Due to these characteristics, aptamers share functional similarities with antibodies, leading to their designation as “chemical antibodies” [1,5,7,8]. Consequently, aptamers are often compared to antibodies and offer various advantages (Table 1). Development and manufacturing of aptamers do not require any biological systems, making the process cheaper, safer, and easier to reproduce, resulting in lower batch-to-batch variation. Additionally, this approach allows for the use of toxic and/or nonimmunogenic targets. Aptamers are less sensitive to pH and temperature changes compared to antibodies, resulting in longer shelf life. Furthermore, their heat-induced denaturation is reversible, unlike proteins, which undergo irreversible denaturation. Aptamers themselves are low-/non-immunogenic, even when exceeding the therapeutic doses, except phosphorothioates (see more below). In addition to these advantages, aptamers also exhibit the beneficial characteristics of antibodies, including specificity, robust binding to targets, and the potential to target a wide (even wider) range of molecules [2,4,6,27,28,29]. One crucial factor is the nucleic acid-based structure of aptamers, which allows complementary oligonucleotides to hybridize with them, thereby hindering their ability to bind to the intended target, enabling the development of specific antidotes against aptamers [3,7,30].

It is worth mentioning that RNA aptamers can also be produced in vivo through direct expression using specific strains of host bacteria, such as *Rhodovulum sulfidophilum*, which can efflux nucleic acids but not RNases. Furthermore, RNA production using tRNA was also employed, and recently, a novel hybrid platform that integrates tRNA and pre-miRNA has been established to facilitate the large-scale production of bioengineered RNA agents including aptamers [31].

The main drawbacks of aptamers stem from their low stability in biological systems, which is due to two main factors. First, because of their oligonucleotide nature, aptamers are degraded by nuclease enzymes. Second, due to their small size, aptamers are rapidly eliminated from the body through renal filtration. However, these issues can be overcome by using modified nucleic acids instead of natural ones (see more below). Another challenge is that because of their polyanionic phosphodiester backbone, aptamers tend to bind more readily to positively charged targets, while negatively charged targets are not preferred due to the electric repulsion between the aptamer and the target. Additionally, a certain concentration of cations is required in the solution to ensure that the aptamers can properly fold into the 3D structure necessary for target binding [2,32]. Another issue is that aptamers poorly penetrate cells, primarily reaching extracellular targets. Intracellular molecules can be targeted with aptamers through proper formulation or by using transient expression. Aptamers designed for intracellular targets are known as intramers [2,7,30].

**Figure 1:**
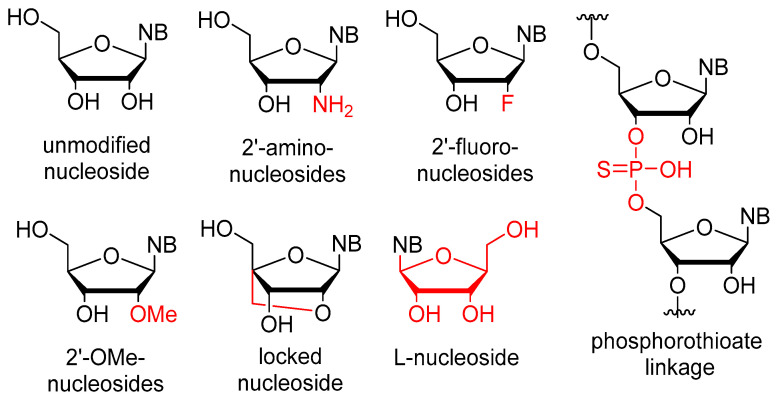
Chemical modifications used in aptamers; differences from the natural nucleosides are highlighted in red.

The stability issues mentioned above can be effectively managed by chemical alterations of the oligonucleotide structure (Figure 1). Between RNA and DNA, the latter offers higher stability, but RNA demonstrates more favorable binding properties owing to its flexibility. As a result, several modified RNA aptamers have been developed. The most common site for modification is the 2′-position of the ribose ring. The modification must fulfill two criteria: (1) increase the stability of the aptamer, and (2) not hinder the binding to the target. It is also important that the modified nucleosides can be used as building blocks by polymerase enzymes for the synthesis of the aptamers. Fortunately, mutant polymerases have been identified, which can accept several modified nucleosides. One of the most important nucleases, RNAse A, exhibits specificity towards pyrimidine nucleosides. In the context of aptamers, the pyrimidine nucleosides are subjected to modification at the 2′-position through the introduction of either amino groups or fluorine atoms. The utilization of an amino group presents certain drawbacks as it necessitates the use of protecting groups during solid-phase synthesis. Moreover, despite increasing nuclease resistance, 2′-amino substitution is associated with a reduction in binding affinity. Conversely, the 2′-fluoro modification does not show these limitations and concurrently ensures increased stability and binding affinity. For the purine nucleosides, the cheaper 2′-OMe modification is commonly used [2,7,30,33,34]. In addition to the ribofuranose moiety, other parts of the molecule can also be modified. The phosphodiester bond can be replaced by a phosphorothioate. While this alteration is prevalent in antisense oligonucleotides, it has the downside of inducing aspecific interactions with proteins, thus rendering it less preferable [32,35]. Nucleosides containing modified bases can also be utilized in aptamers. Additional modifications, such as the incorporation of locked nucleosides (LNA) or L-nucleosides (Spiegelmer, derived from the German word “spiegel” meaning mirror), are less common [35]. Aptamers frequently feature a “capped” 3′-end, where inverted deoxythymidine is linked to the aptamer via a 3′-3′ bond instead of the normal 5′-3′, providing further resistance against nucleases. Truncation is a process in which the length of the oligomer selected through SELEX is decreased until the shortest efficient aptamer is obtained. This helps to decrease potential unintended bindings and reduces the cost of synthesis [32]. To enhance stability, it is necessary to not only increase nuclease resistance but also reduce renal clearance. To achieve this, the aptamer is conjugated with cholesterol, IgG, or most frequently, polyethylene glycol (PEG) [2,7,32].

Aptamers serve various functions, including acting as delivery agents, diagnostic tools, and therapeutic agents [3,4,27,29,36]. They can be used as medicinal agents for combating protozoal infections and cancer [4,27,37]. Notably, aptamers have been designed to target a range of proteins, such as coagulation factors IXa and IIa (thrombin), von Willebrand factor (vWF), nucleolin, human epidermal growth factor receptor 2 (EGFR-2, HER-2), protein kinase 7, cytotoxic T cell antigen 4, angiopoietin-2, etc., [. Vascular endothelial growth factor (VEGF) is a glycoprotein that plays a pivotal role in angiogenesis. VEGF is active in a disulfide-linked homodimer form and causes the proliferation and migration of endothelial cells. Due to alternative splicing and post-translational proteolytic processing, VEGF exhibits several isoforms with different solubility, heparin-binding properties, and receptor affinity, consequently having distinct biological roles based on their interactions with different receptors. Given this diversity, the inhibition of all isoforms is unadvisable due to the potential disruption of a wide range of critical biological processes. Exons 6 and 7 encode the basic amino acid-rich heparin-binding domains of the VEGF. The 189 and 206 isoforms encompass both domains, resulting in strong binding to heparin. Conversely, VEGF-165 contains only exon 7, leading to weaker heparin binding, thereby promoting enhanced solubility and improved diffusion within tissues. In contrast, the 121 isoform does not have a heparin-binding domain (HBD), leading to superior solubility and diffusibility. However, this isoform also exhibits lower receptor affinity, consequently manifesting weaker mitogenic activity. VEGF is implicated in the pathogenesis of several ocular disorders, including age-related macular degeneration (AMD) and diabetic retinopathy (DR). AMD is a degenerative retinal disease and the leading cause of blindness in developed countries, with the highest prevalence among Europeans compared to African, Hispanic, and Asian populations [26,38]. In 2020, the estimated global population of individuals affected by AMD was 196 million. This number is projected to rise to 288 million by 2040, mainly due to longer life expectancies and the adoption of the Western diet and lifestyle [39]. AMD manifests in two forms: the dry (atrophic) form and the wet or exudative (neovascular) form. Currently, there are no available treatments for dry AMD, which is characterized by the thickening of Bruch’s membrane (BrM) resulting from the accumulation of lipids and proteins. This process leads to the formation of sub-retinal pigment epithelium (RPE) deposits called drusen. The presence of drusen disrupts the fluid efflux from the RPE across Bruch’s membrane, causing both chemical and mechanical separation between the RPE and the choroid, thereby reducing the perfusion to the RPE [38]. In neovascular AMD, abnormal choroidal neovascularization (CNV), which is the hallmark of this form, is developed in approximately 10% of AMD patients and results in complications such as leaking, hemorrhage, detachment of RPE, and hard exudate deposition, ultimately causing central vision loss. Notably, the neovascular form accounts for 80–90% of AMD-caused vision loss. Given that the prevalence of AMD rises with age, it poses an increasingly serious concern in developed countries with aging populations [33,40,41,42,43].

VEGF appears to be critical for the development of CNV. VEGF-165 isoform is the main driver of inflammation and cellular immune responses associated with pathological retinal neovascularization. It functions as a pro-inflammatory cytokine, which targets monocytes, macrophages, and leukocytes, establishing a positive feedback loop predominantly involving endothelial cells (ECs) and promoting the neovascularization process [44,45]. VEGF isoforms expression pattern is tightly regulated under normal conditions. However, this regulation is often disrupted in diseases. In vivo studies in rodents have shown a ten-fold increase in VEGF-165/VEGF-120 ratio, reaching ~25.5 in pathological retinal neovascularization, compared to ~2.2 in a physiologically developing retina. This change likely triggers an angiogenic switch and contributes to inflammation-associated vessel invasion within the vitreous [44]. Therefore, VEGF emerges as an excellent target for treating AMD or DR.

Pegaptanib, the first approved aptamer, and the foremost anti-VEGF medication, was granted FDA approval in 2004 for the treatment of AMD. As we mark its 20th approval anniversary, we herein present a brief historical overview of pegaptanib. In addition, we cast an eye over the therapeutic competition between pegaptanib and monoclonal antibodies, while also evaluating the current status of aptamers in the treatment of AMD, placing particular emphasis on avacincaptad pegol, the second aptamer recently approved by the FDA.

## 2. Pegaptanib

### 2.1. Chemistry and Mechanism of Action

**Figure 2:**
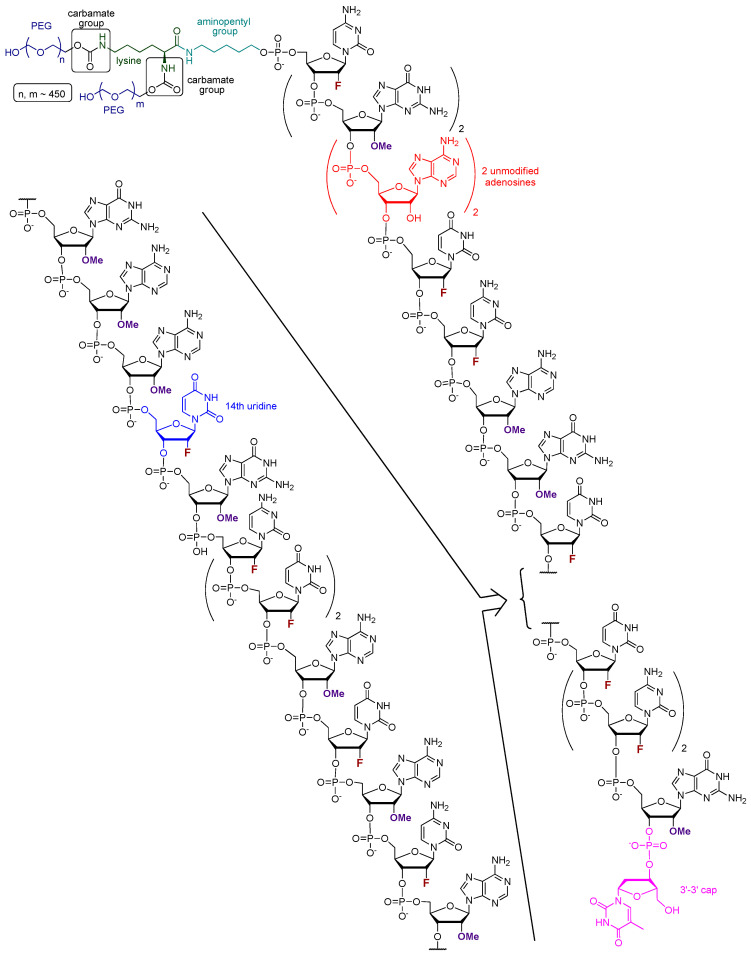
The structure of pegaptanib; different types of synthetic modifications are highlighted using different colors.

Pegaptanib (tradename Macugen, in early publication also referred to as t44OMe, NX1388, or EYE001) is an RNA aptamer with the sequence of 5′-CGG AAU CAG UGA AUG CUU AUA CAU CCG-3′-3′dT (Figure 2). All pyrimidine nucleosides are 2′-deoxy-2′-fluoro nucleosides, granting higher nuclease resistance and favorable binding properties to the molecule. The purine nucleosides, except for the 4th and 5th adenosines underlined in the sequence, are 2′-OMe substituted. To the 3′ end, a deoxy-thymidine is connected to the final guanosine via an inverted 3′-3′ phosphodiester bond, providing protection against exonucleases. At the 5′ terminus, a lysine is connected to the oligonucleotide via an aminopentyl group. Additionally, two polyethylene glycol monomethyl ethers, with the size of 20–20 kDa, are linked to the lysine through carbamate bonds [35,42,46].

**Figure 3:**
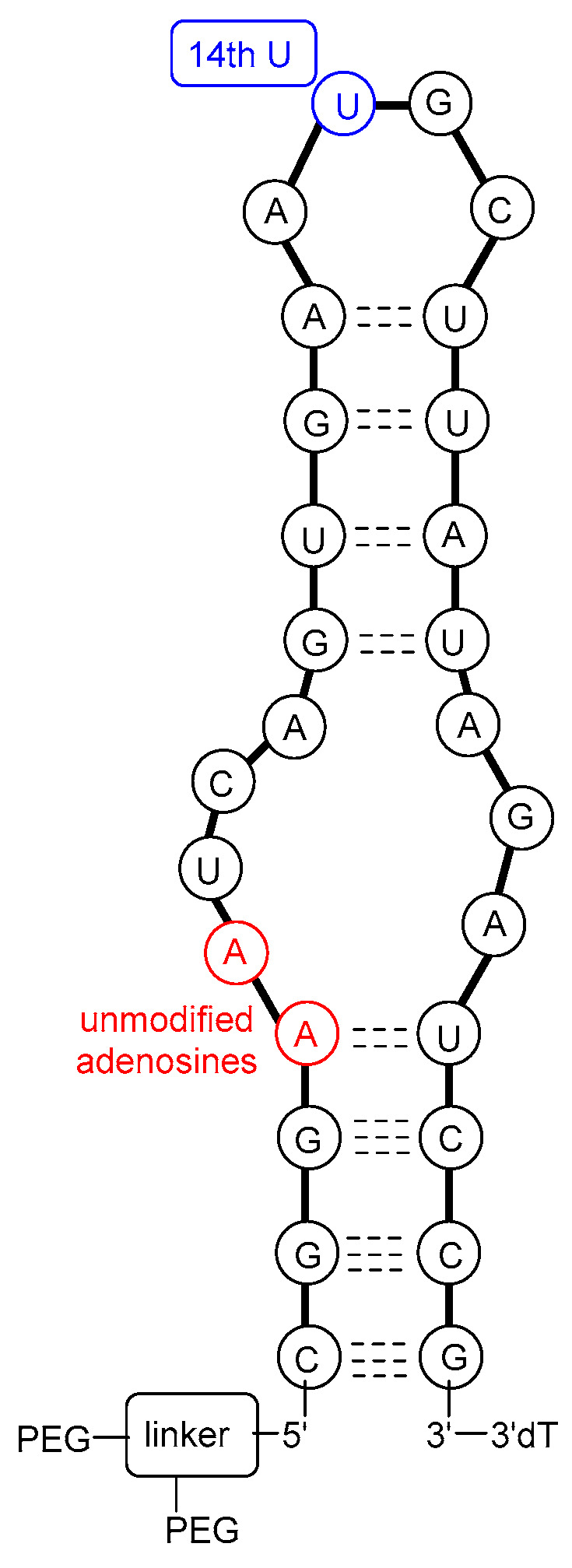
A possible secondary structure of pegaptanib.

The molecular target of pegaptanib is VEGF. It binds to the exon 7-encoded heparin-binding domain, with an extremely high affinity (K_d_ = 50 pM) [47], rendering it selective to the 165 isoform while displaying no binding affinity for the 121 isoform which lacks this domain. Pegaptanib’s binding to VEGF-165 inhibits its interaction with its receptors, VEGFR1 and VEGFR2, and natriuretic peptide receptor 1 (NPR1), thereby blocking its biological activities [48]. In experiments using cultured human umbilical vein endothelial cells (HUVEC), pegaptanib effectively inhibited the binding, signal transduction, calcium mobilization, and cell proliferation induced by VEGF-165, to a degree comparable to that observed with an anti-VEGF monoclonal antibody [49]. Some reports show that pegaptanib exhibits a lower, yet significant, affinity for binding to VEGF-189 compared to VEGF-165. Pegaptanib’s binding to exon 7-containing VEGF183 and VEGF206 may also be possible, although these isoforms are expressed at very low levels and play a minimal role in angiogenesis [48]. The 14th uridine of pegaptanib can undergo photo-crosslinking with Cys-134 of VEGF-165 (Cys-27 of the heparin-binding domain), proving the binding location (Figure 3) [42,46,50,51,52].

### 2.2. Development and Approval

#### 2.2.1. Chemical Development and In Vitro Assays

In 1994, it was first reported that RNA ligands are capable of binding to VEGF. Researchers examined the sequence–activity relationship and truncated oligomers and found that certain aptamers can inhibit the interaction between VEGF and its receptor within the range of 20–40 nM half inhibition cc. This study laid the foundation for further research toward developing more potent and stable molecules [53]. In 1995, the discovery of VEGF-binding aptamers consisting of 2′-aminopyrimidines was reported. Following SELEX, sequences demonstrating the highest affinity were selected. Subsequently, post-SELEX modifications were conducted to investigate which purines can be substituted in the 2′-O position with a methyl group without weakening VEGF-binding ability. Notably, in some positions, the 2′-OMe modification not only enhanced nuclease resistance but also significantly improved the binding affinity with a 17-fold increase observed, while in other positions, this modification exhibited either a neutral or detrimental effect on binding. Additionally, short phosphorothioate sequences were introduced at the 3′ and 5′ ends to confer further protection against exonucleases [54]. The direct antecedent of pegaptanib was the work of Ruckman et al. in 1998 [50]. As mentioned previously, the 2′-fluoro modification is more advantageous than the 2′-amino modification. As a result, 2′-fluoro pyrimidine nucleoside-modified aptamers were developed through SELEX. Ten SELEX cycles increased the affinity of the oligonucleotide pool in a 1000-fold manner. However, two additional cycles did not further improve the affinity, so the sequences from the 12th cycle were used for further development. Out of the 143 isolated clones, 46 sequences were categorized into three families. Selected members of these families were truncated to identify the minimal binding sequence. Subsequently, the effect of 2′-OMe incorporation was evaluated for 3 selected truncated compounds. The development stages of the pegaptanib oligonucleotide “core” were as follows. VT30.44 was isolated from the third family of SELEX-processed oligonucleotides, where “V” stands for the VEGF as the molecular target, “T” is for the TBS (tris buffer saline) buffer used for the selection, “30” represents the length of the randomized region in the library, and “44” indicates the number of the clone. The t44.2 is the truncated (27 nucleotide long) version of VT30.44, as the “t” indicates. The t44-OMe contains OMe nucleotides in all positions, except for the 4th and 5th, as this structural change in these two adenosines causes a significant drop in the binding affinity. It was demonstrated that the presence of Ca^2+^ ions is necessary for high binding affinity. The binding affinity and selectivity were examined and it was proven that t44-OMe binds with high affinity to human and mouse VEGF-165 while showing no binding to VEGF-121. Photo-crosslinking reactions were performed by changing 1-1 2′-deoxy-2′-fluorouridine residue to 5-iodouridine in specific positions. The most efficient reaction was observed when the 14th uridine was modified. Using this method, the aptamer binding was mapped to Cys-137 of the VEGF, indicating that the binding occurs in close proximity to that position. The compound t44-OMe inhibited the binding of VEGF-165 to FMS-like tyrosine kinase-1 (Flt-1) and kinase insert domain-containing receptor (KDR) receptors (VEGFR1 and VEGFR2, respectively). Among the tested compounds, t44-OMe emerged as the most potent inhibitor of VEGF-mediated vascular permeability. Conjugation of a 40 kDa PEG to the 5′ end reduced binding to the target but increased the inhibitory activity [50]. The PEGylated version of t44-OMe featuring an inverted 3′-3′ deoxythymidine at the end was later denoted by the code NX1838.

#### 2.2.2. Preclinical Studies

An HPLC method for detecting NX1838 in the plasma was developed in rhesus monkeys. A dose of 1 mg/kg NX1838 was administered either intravenously (i.v.) or subcutaneously (s.c.), and the main plasma pharmacokinetic parameters were determined. In the i.v. group, the Cmax was 25.5 μg/mL with an elimination half-life of 9.3 h. In the s.c. group, the Cmax was 4.9 μg/mL with a 12 h elimination half-life. The area under the curve (AUC) of the s.c. group was 78% relative to the i.v. group, suggesting that most of the active ingredient was absorbed into the systemic circulation. These results are similar to the observations with rats. It is important to note that the concentration of NX1838 remained over 15 nM for 3 days, which is significantly greater than the estimated KD of NX1838 for VEGF-165 (~200 pM) [46]. The research conducted on HUVEC demonstrated that NX1838 inhibits the binding of VEGF to the cells, VEGF-dependent phosphorylation of KDR and phospholipase C gamma (PLCγ), and VEGF-induced calcium mobilization [51]. To further support the clinical development, the intravitreal pharmacokinetics of NX1838 was assessed in rhesus monkeys. It was determined that the active ingredient was eliminated from the vitreous humor to the plasma intact with a half-life of approximately 94 h (depending on the dose used), which is similar to the findings observed in rabbits (83 h). The plasma terminal half-life ranged between approximately 90–100 h (depending on the dose used). Considering that, in the case of intravenous injections, the terminal plasma half-life is 9.3 h, the rate-determining step is probably the clearance from the eye to the plasma. After 28 days, the active ingredient in the vitreous humor is still fully capable of binding to VEGF. Even in the lowest dose receiving group (0.5 mg/eye), the concentration of NX1838 in the vitreous humor after 28 days was 170 ± 7 nM. This is important because, as mentioned earlier, the KD value of the VEGF binding is 200 pM, while the VEGF concentration in the vitreous humor is less than 2 nM. Based on this, it was suggested that a monthly dose of 1–2 mg/eye/month dose could be efficient for treating human patients (taking into account the larger volume of vitreous humor in humans) [55,56].

Other preclinical experiments were also conducted. In guinea pigs, compound NX1838 (coded as EYE001) almost completely inhibited VEGF-induced vascular leakage, as measured by the Miles assay (the leaking of Evans blue dye from the vasculature was determined after the administration of VEGF). Neovascularization in the cornea was decreased by 65% by EYE001. In the retinopathy of prematurity (ROP) mouse model, 80% inhibition of neovascularization was observed. In vivo, Compound EYE001 also inhibited the growth of A67 rhabdomyosarcoma tumor xenografts in mice. It is important to note that in these cases, the drug was systematically administered [56]. In the study, conducted by Ishida et al. [57], diabetic rats received intravitreal injections of VEGF and EYE001 (2.5 nmol). The aptamer reduced VEGF-induced early diabetic retinal leukostasis by 72% and established diabetic retinal leukostasis by 48%. Furthermore, the treatment inhibited early blood-retinal barrier breakdown (BRB) by 83%, and the established BRB was inhibited by 55%. These findings indicate that although the drug was designed against human VEGF-165, it effectively mitigates the effects of rat VEGF-164 [57]. Carrasquillo et al. reported the development of a controlled delivery system for the EYE001 based on the biodegradable polymer poly(lactic-co-glycolic) acid (PLGA). The encapsulated active ingredient was released from the microspheres over 20 days while retaining its activity [58].

#### 2.2.3. Clinical Studies

In 2002, the first clinical results were published. 15 patients were treated in a clinical phase 1A open-label study. The participants were 50 years old or older patients with subfoveal CNV, with best corrected visual acuity (VA) worse than 20/200 (measured with ETDRS = Early Treatment Diabetic Retinopathy Study charts), including at least 1 patient in each cohort with worse than 20/400. A single dose of EYE001 was administered by intraocular injection. Applied doses ranged between 0.25 and 3.0 mg/eye. Dose-limiting toxicity was not observed. After 3 months, 80% of patients showed stable or improved vision, and 27% showed significantly improved vision (three lines or greater increase in vision, based on ETDRS chart). Reported adverse events were mild to moderate and included generic side effects such as fatigue, as well as those probably related to the route of administration such as eye pain or intraocular inflammation. In summary, the safety of EYE001 was confirmed. However, a serious limitation of the study is the small sample size and the lack of a control group [56]. Adverse events reported were mild to moderate and included general side effects such as fatigue, as well as those related to the route of administration, such as eye pain or intraocular inflammation.

Phase II results were published in 2003. The study involved 21 patients who were treated with multiple doses of 3 mg of EYE001 every 28 days for a total of 3 times (in this study, EYE001 was sometimes referred to as pegaptanib). The patients had subfoveal CNV (secondary to AMD) with a VA of the study eye worse than 20/100 and better or equal to 20/400 of the fellow eye, based on ETDRS chart. The patients received either pegaptanib alone or pegaptanib together with photodynamic therapy (PDT). In the pegaptanib group of 8 patients, after completing the treatment, 87% of patients had stabilized or improved vision and 25% had a 3-line improvement on ETDS charts. In the pegaptanib + PDT group of 10 patients, 90% had stabilized or improved vision, and 60% had a 3-line improvement. No serious adverse effects linked to pegaptanib were observed. Non-serious adverse events included mild anterior chamber inflammation, eye pain, ocular irritation, increased intraocular pressure, dry eye, and other local reactions, possibly due to the administration route. This study has the limitations of the lack of a control group, a small sample size, and a limited follow-up period. However, historical data suggests that PDT alone is significantly inferior to both pegaptanib alone and pegaptanib combined with PDT. Additionally, the results are consistent with the earlier phase I study [59].

In parallel with the previous study, a guest editorial was published in the same journal, highlighting the potential risks of such an anti-VEGF therapy. It was stated that following intravitreal injection, the active ingredient is absorbed into the systemic circulation in its active form. Due to its high inhibitory potency, even a low concentration of the active ingredient can interfere with VEGF-mediated biological processes. Of particular concern is the potential inhibition of cardiac neovascularization after ischemia, especially among elderly patients who are also the main target population for pegaptanib, as AMD predominantly manifests in old age [60].

In 2004, the results of a double-blind multicenter randomized controlled trial (RCT) with more participants and a longer follow-up period (V.I.S.I.O.N. trial) were published. The trial involved 1186 patients receiving either 0.3 mg (n = 297), 1.0 mg (n = 305), or 3.0 mg (n = 302) of pegaptanib, or a sham injection (identical injection but without a needle pressed against the eye wall, n = 304) every 6 weeks over 48 weeks (9 doses). The participants were 50 years old or older and had subfoveal CNV secondary to AMD, with a best-corrected VA of 20/40 to 20/320 in the study eye and at least 20/800 in the fellow eye. The primary endpoint was the ratio of patients who lost < 15 letters of VA at week 54. The results were 70%, 71%, and 65% in the 0.3 mg, 1.0 mg, and 3.0 mg pegaptanib groups, respectively, while this ratio was 55% in the sham injection group. These results suggest that higher pegaptanib doses have no significant benefit. Regarding the secondary endpoint, the percentage of patients who gained or maintained VA were as follows: In the pegaptanib groups, 33% (0.3 mg), 37% (1.0 mg), and 31% (3.0 mg), while it was 23% in the sham injection group. Within the pegaptanib groups, the ratio of patients who experienced a gain of 15 or more letters was 6% (0.3 mg), 7% (1.0 mg), and 4% (3.0 mg), whereas in the sham injection group, this value was 2%. It is important to note that the risk of severe vision loss (loss of 30 or more letters) was 10% in the 0.3 mg group, 8% in the 1.0 mg group, and 14% in the 3.0 mg group, while in the sham injection group it was 22%. This means that in each treatment group, the risk of severe vision loss was approximately half that of the sham injection group. In the pegaptanib treatment groups, the size growth of the lesion and CNV were also decreased, as well as the leakage. Most adverse events, such as eye pain, anterior chamber inflammation, and corneal edema, were mild to moderate and likely related to the route of administration rather than pegaptanib. The most serious adverse events observed were endophthalmitis (1.3% of patients), traumatic lens injury (0.7%), and retinal detachment (0.6%). In summary, the benefit of pegaptanib was shown to be statistically significant and clinically meaningful for patients with neovascular AMD [61]. The results are promising; however, some questions have arisen. For instance, no dose–effectiveness relationship could be detected based on the data. Furthermore, even the highest dose appears to be less active in certain aspects. One possible explanation is that all three chosen doses are in the “plateau” region of the dose–response curve, which aligns with previous clinical and preclinical findings. Additionally, pegaptanib’s high affinity for VEGF allows for a high effect at low concentrations. Another issue is that there is a difference in lesion size between the baseline of the 0.3 mg pegaptanib group and the sham injection group, favoring the former. This can be a potential con-founder in the trial as lesion size might influence disease progression. It is important to note that while pegaptanib slowed down lesion growth, it did not stop it [61,62]. Subgroup analysis revealed that the 0.3 mg pegaptanib group did not show a statistically significant difference compared to the sham, regardless of whether it was predominantly classic lesions or occult without classic lesions. Moreover, lesions with ≥4 disk areas showed no significant efficacy, highlighting the importance of lesion size. However, the significance of these subgroup analyses is debatable when considering the overall picture. Some critics questioned the study’s use of mean loss of letters instead of the more conventional 3-line response rate. However, these critics had no doubts about the efficacy of pegaptanib [63,64].

**Figure 4:**
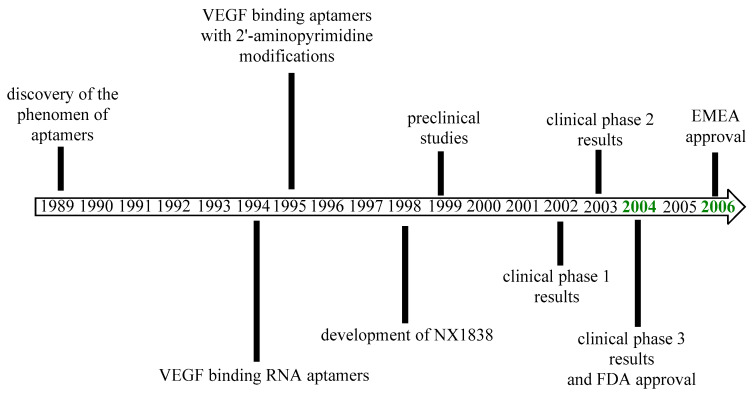
Development of pegaptanib.

Pegaptanib was approved by the FDA in December 2004 for the treatment of neovascular AMD as the first aptamer and also the first anti-VEGF medicine to receive approval [65,66]. The EMA approved pegaptanib in January 2006 [67]. The most important milestones in the history of pegaptanib are outlined in Figure 4. The efficacy results of pegaptanib are summarized in Table 2.

### 2.3. After Approval

#### 2.3.1. General Information

The history of pegaptanib sodium did not end with its approval. Safety data collection continued for several years, yielding long-term safety results. The most common or potentially serious adverse effects were examined in more detail. The efficacy was also observed in extended studies and tested for other indications, demonstrating pegaptanib’s potential in treating various diseases, including relatively common conditions, such as diabetic retinopathy, or rare disorders, such as von Hippel Lindeau disease. Additionally, the cost-effectiveness of pegaptanib was also evaluated. Studies on its combination with other medicines and comparison with antibodies were also reported.

Below, we briefly summarize the studies reported after the 2004 approval of pegaptanib.

#### 2.3.2. Efficacy

##### Age Related Macular Degeneration (AMD)

The V.I.S.I.O.N. trial was extended for an additional year following a re-randomization of patients after the 54th week. The continued treatment was found to be beneficial for the patients. Those who received pegaptanib for two years experienced an average loss of 9.4 letters on ETDRS chart, while the sham group lost 17 letters. The one-year treated group had 35 instances of 3 or more line loss, compared to 21 in the two-year treated group. The mean VA remained stable in the group that continued receiving pegaptanib during the second year, while it decreased in the group that stopped receiving pegaptanib or did not receive it at all. The difference between the one-year-treated and non-treated groups suggests that pegaptanib not only reduces symptoms but also has a disease-modifying effect. Pegaptanib was well-tolerated in the second year as well [65,68,69,70,71,72,73]. Analysis of a subgroup of patients showed that pegaptanib treatment was associated with an improvement in vision-related quality of life (VRQoL) [74].

In a previous study, patients mainly had progressed disease. However, it was found that pegaptanib treatment could be more effective if started in an earlier stage of the disease. An extrapolatory analysis was conducted with early-disease subgroups of the 0.3 mg pegaptanib group from a previous study [61] to examine the benefit of pegaptanib in early subfoveal CNV. Patients were divided into two groups. In the first group, patients with a lesion size of <2 disk areas, baseline VA ≥ 54 ETDRS letters, no scarring or atrophy in the lesion, and no prior PDT or thermal laser photocoagulation to the lesion were included. In this group, 34 patients received 0.3 mg pegaptanib, and 28 patients received a sham injection. In the second group, the criteria were: occult with no classic CNV, absence of lipid, and better VA at baseline in the fellow eye. In this group, 30 patients received 0.3 mg of pegaptanib, and 35 patients had sham injections. Baseline characteristics were well-balanced in both groups. The primary endpoint was the loss of less than 15 letters of VA. In the first group, 76% of the patients treated with pegaptanib showed a positive response in the primary endpoint, while only 50% of the patients in the sham injection group showed a response. The average change in VA was −5.6 letters for the pegaptanib group and −16.6 for the sham injection group. Among the sham injection group, 29% of subjects experienced severe vision loss, while in the pegaptanib-treated group, only 3% did, which is a tenfold difference. Additionally, there was a higher chance of maintaining or gaining vision in the pegaptanib-treated group compared to the sham injection group. In the second group, the response rate for the primary end-point was 80% and 57% in the pegaptanib-treated and sham injection groups, respectively. The average change in VA was −4.0 letters for the pegaptanib-treated group and −16.7 letters for the sham injection group. The likelihood of severe vision loss was 23% in the sham injection group and 10% in the pegaptanib group. In the group of patients treated with pegaptanib, a higher proportion of patients maintained or gained vision. These findings indicate that, as expected, early treatment with pegaptanib is associated with better clinical outcomes, possibly due to the preservation of photoreceptors resulting from early intervention in the progression of the disease [75]. The data from 90 patients with newly diagnosed neovascular AMD was reviewed retrospectively with a follow-up period of 6–14 months. Among the patients, 20% gained ≥3 lines of vision, 60% maintained their vision (losing less than 3 lines), and 10% lost more than 3 lines. The mean visual acuity improved from 20/100 to 20/80. In 33 patients, the central retinal thickness was measured and it was found to be changed from 295.4 ± 85.2 μm to 277 ± 78.2 μm. These findings suggest that pegaptanib may be more beneficial in naïve CNV. Limitations of this study include its retrospective, uncontrolled, and nonrandomized nature [76].

A case report was published about two patients experiencing AMD-related CNV progression 6 weeks after receiving a single dose of pegaptanib. This does not contradict the findings of previous phase III clinical trials in which pegaptanib was administered multiple times, and angiographic evaluations were conducted at 30 and 54 weeks. However, it points out that monitoring CNV progression is recommended after a pegaptanib injection [77]. Another study suggests that the benefits of pegaptanib may not be evident in the short term after a single injection. The study involved 29 patients with neovascular AMD who were administered 0.3 mg of pegaptanib. Central foveal thickness (CFT) was measured by optical coherence tomography (OCT). Before receiving pegaptanib, the mean CFT was 343 ± 117 µm, with a range of 187–795 µm. After 6 weeks, the mean CFT was 334.3 ± 101.3 µm, with a range of 173–648 µm, indicating a nonsignificant 0.86% change. It is important to note that, in some cases, up to a 70% increase in CFT was observed. Additionally, the VA of the patients did not show significant improvement, with 69% maintaining stable VA, 3.4% gaining ≥2 lines, and 27.6% losing ≥2 lines. The study found that neither the baseline characteristics nor the subtype or size of the lesion influenced the outcomes [78].

A similar study was conducted with more patients (n = 41), and over a longer follow-up period (12 weeks). The dose used (1.0 mg every 6 weeks) was higher than the previous study’s dose of 0.5 mg. However, previous studies (see more above) suggest that the higher dose should not significantly affect the outcome. The baseline CFT, measured by OCT, was 340 ± 24 μm, similar to the previous study. At week 6, the CFT decreased to 312 ± 16 μm, resulting in a statistically insignificant decrease in thickening of 22% (*p* = 0.3). By week 12, the CFT further decreased to 299 ± 14 μm (32% decrease). Initially, the fluorescein angiographic leakage was observed in 100% of the patients and was decreased to 81% by the 12th week. 31 patients continued the study for 6 months, and at that point, their VA remained stable. 54% of patients still had definite leakage, and the CFT reduced to 274 ± 14 μm, marking a 51% reduction. These findings indicate that pegaptanib can reduce central retinal thickness, but the effect takes more than 6 weeks to develop [79].

14 patients with AMD-related CNV received 0.3 mg pegaptanib every 6 weeks for an average of 30.5 ± 8 weeks. In this study, 29% of the patients showed at least a 1-line VA improvement, 21% remained stable, and 50% showed at least a 1-line decrease in VA. CFT changed from 383 ± 83 μm to 407 ± 116 μm. The study’s limitations were the small sample size and the lack of a control group [80].

In a study involving 49 patients for 6 months, it was found that 90% of treated eyes maintained their VA (with less than 3 lines loss or gain). Only 2% of the patients experienced a loss of 3 or more lines, while 8% experienced a gain of 3 or more lines. The CRT changed minimally from 251.19 ± 75.83 μm to 251.63 ± 68.23 μm, and no serious complications were reported. However, It is important to note that no control group was included in this study [81]. In a separate German study, 27 patients were treated with pegaptanib. 89% of the patients lost 3 or fewer lines over 24 weeks, while 33% experienced a gain in VA. Only one complication, endophthalmitis, was reported [82].

In one study, 43 patients were given intravitreal pegaptanib for 12 months. The baseline VA, measured using the logMAR (logarithm of the minimum angle of resolution) on ETDRS chart, was 1.25 ± 0.43, and the foveal thickness was 452.3 ± 44.83 μm. After 12 months, VA improved to 0.83 ± 0.44 (*p* = 0.03), and none of the patients showed any deterioration. Additionally, the foveal thickness decreased to 274.3 ± 13.33 μm [83].

The records of 73 patients with neovascular AMD were retrospectively reviewed. The patients were given at least 4 doses of pegaptanib over 21 weeks, with 6-week intervals between doses. The baseline VA was 0.62 ± 0.24 (logMAR) or 20/80 (Snellen). By month 6, 70% of patients had lost less than 3 lines of vision (the average change was −0.63 lines or −0.07 logMAR). It was observed that 2 subgroups of patients with early-stage disease had a more positive response to pegaptanib compared to the entire group [84].

A retrospective chart review was conducted on AMD patients with RPE tears. Only 5 patients were included, with a median baseline VA of 20/200, which remained stable in the 12th month and improved to 20/60 in the 24th month. However, due to the small sample size, the results were not significant [85].

In a European study, data from 253 patients treated with pegaptanib over 24 weeks underwent retrospective review. At week 24, 93% of the patients lost less than 15 letters. Among the 62 patients who participated in the extended follow-up, 92% lost less than 15 letters in week 54. Furthermore, 73% and 71% maintained or gained vision in weeks 24 and 54, respectively [86].

In a study involving 40 patients, pegaptanib was administered every 6 weeks for a total of 3 treatments. The baseline VA was 0.99 ± 50 (logMAR), which changed to 0.97 ± 0.39 (non-significant). Notably, there was a significant change in retinal thickness from 432.6 ± 164.3 to 359.0 ± 132.1 μm. Additionally, 76% of the treated eyes exhibited either stable or improved VA [87].

23 patients with subfoveal CNV secondary to AMD were treated. 12 patients had a history of cerebrovascular accident, and 11 had myocardial infarction. The best-corrected VA logMAR was 0.67 ± 0.23 (~20/100 Snellen equivalent) at baseline, and CFT was 381 ± 111 µm. By the 12th month, the VA improved to 0.52 ± 0.31 logMAR (~20/40 Snellen equivalent). 35% gained at least 3 lines, 48% remained stable, while 17% lost 3 or more lines. CFT decreased to 304 ± 82 µm. No systemic or local side effects were detected, and the patients did not experience further arterial thromboembolic events (ATE) [88]. This is an important result as it demonstrated that pegaptanib can be effective and safe for treating patients with ATE, addressing a previous concern about anti-VEGF therapy [60]. However, the results are limited by the small sample size and the lack of a control group.

In a study involving 56 patients with occult CNV, 8 pegaptanib injections were administered at 6-week intervals. By the 52nd week, 79% of the patients lost less than 15 letters of VA. Additionally, central retinal thickness decreased by 102 μm at week 52, compared to the baseline measurement of 262–364 μm [89].

**Figure 5:**
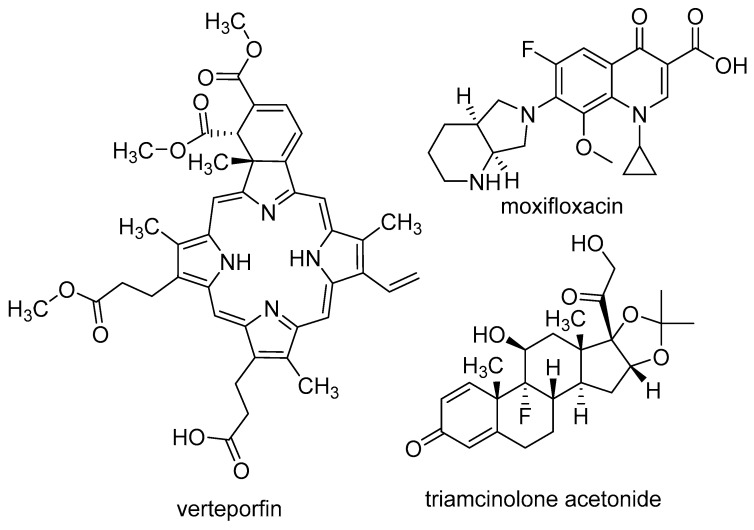
Structures of medications used in combination with pegaptanib.

The investigation encompassed not only the singular effect of pegaptanib but also its potential combination with other medications (Figure 5). One possible approach involves combining pegaptanib with PDT, which was used to treat AMD before the approval of pegaptanib. PDT is based on the use of verteporfin, which accumulates in choroidal neovasculature. When photoactivated, verteporfin generates reactive oxygen species (ROS) that damage the endothelial cells. This causes the formation of a “plug” by the thrombocytes, ultimately leading to the occlusion of the vessel. since verteporfin PDT targets the vascular component of the disease, while anti-VEGF agents target the angiogenic component, it seems reasonable to expect some synergism from combining these two types of treatment [90]. In the phase II study of pegaptanib (see more above), the pegaptanib + PDT group showed better results than the pegaptanib group [59]. In a retrospective interventional case series study, medical charts of 16 patients were reviewed, involving 22 studied eyes. 13 eyes (old treated group) were treated with high-dose intravitreal triamcinolone acetonide and photodynamic therapy (HDIVTA + PDT), while 9 eyes did not receive previous treatment (newly treated group). Triamcinolone was administered, then after 2 weeks, verteporfin-based PDT, and after another 2 weeks, pegaptanib. The baseline mean VA was 20/160. In the newly treated group, the mean improvement was 2.2 lines, 33% of eyes showed at least 3-line improvement, 56% had stabilized VA, and 11% lost 3 or more lines. In the old treatment group, the mean VA change was 0.7 lines, 7.7% of eyes gained 3 or more lines, 61% maintained VA, and 30% lost 2 or more lines. These results suggest that this “triple therapy” is more effective than the single component; however, the results were just compared to literary controls [91]. In a prospective, non-comparative case series, 7 patients with predominantly classic juxtafoveal CNV were included in a 24-week follow-up study. They were administered pegaptanib 48 h after PDT. The mean VA decreased from 60.4 ± 13.2 to 55.2 ± 24.9, which was statistically significant. The total area of CNV increased from 1.4 ± 1.6 mm^2^ to 2.7 ± 2.4 mm^2^. This study suggests that predominantly classic juxtafoveal CNV exhibits a low response to the pegaptanib + PDT combination. However, due to the small sample size, the significance of the results obtained is limited [92]. A total of 45 patients were divided into three groups. Group 1 received pegaptanib + low-fluence PDT, group 2 received pegaptanib + PDT, and group 3 received pegaptanib monotherapy. The central macular thickness (CMT) in group 1 changed from 396.13 ± 119.93 μm to 345.67 ± 74.61 μm, in group 2 from 450.40 ± 230.47 μm to 339.07 ± 129.24 μm, and in group 3 from 422.13 ± 155.58 μm to 348.53 ± 88.95 μm. The decrease in VA was 0.3, 1.0, and 2.2 lines in the respective groups. Additionally, the VA-based treatment success rate (the ratio of patients who lost less than 3 lines) was 86.7%, 80%, and 60% for groups 1, 2, and 3, respectively. These findings suggest that the groups receiving PDT + pegaptanib demonstrated superior outcomes compared to that undergoing pegaptanib monotherapy [93].

Another possibility is to combine pegaptanib with anti-VEGF antibodies. In a pilot study, 48 patients were treated with bevacizumab (n = 13), pegaptanib (n = 18), or bevacizumab followed by pegaptanib (n = 17). Interestingly, bevacizumab monotherapy demonstrated greater efficacy compared to the two other treatment regimens. Within the bevacizumab group, 38.5% of patients gained letters and 61.5% remained stable VA. In the pegaptanib group, 5.5% gained letters and 55.5% remained stable, while 38.9% had decreased VA. In the sequential treatment group, 76.5% maintained VA, while 23.5% experienced a decrease. Contrast sensitivity increased in the bevacizumab group after 6 weeks, decreased in the pegaptanib group after 12 weeks and 6 months, and did not change significantly in the sequentially treated group. Furthermore, macular thickness decreased in all groups [94]. In a phase IV prospective uncontrolled exploratory study, 568 patients who had received 1–3 previous treatments (as induction, bevacizumab, ranibizumab, pegaptanib, PDT or transpupillary thermo-therapy, or a combination of these) 30–120 days before starting pegaptanib treatment (0.3 mg every 6 weeks for 48 weeks, with a follow-up of 54 weeks) were included. The average VA improved during the induction phase from 49.6 ± 21.9 letters to 65.5 ± 15.3 letters. At week 54, the average VA was 61.8 ± 18.9 letters, indicating that pegaptanib treatment successfully maintained the improvements observed [95]. Similar results were reported in a smaller (75 patients) Japanese study. The mean VA (logMAR) was 0.61 ± 31 before induction, 0.26 ± 0.24 after the induction therapy, and 0.29 ± 0.28 after the maintenance therapy (over 54 weeks) [96]. Another study in Japan involved 19 patients (20 eyes studied) with previously untreated AMD who received ranibizumab for 3 months as induction, followed by pegaptanib as maintenance therapy every 6 weeks, with a long, 3-year follow-up. The mean logMAR VA at the baseline was 0.56 ± 0.31, which improved to 0.24 ± 0.25 after induction, and to 0.25 ± 0.28 at the 156th week. Additionally, baseline CFT was 346 ± 111 µm, which reduced to 232 ± 54 µm after induction and further reached 210 ± 59 µm after the maintenance therapy [72]. This approach of using MAB as an induction therapy, followed by pegaptanib for a longer term as maintenance, can be advantageous. This is because antibodies are generally more effective than pegaptanib (see more below), but they also non-selectively target all VEGF isoforms, whereas pegaptanib selectively targets the 165 isoform responsible for pathological neovascularization in the eye. Therefore, the rationale behind this combination is to achieve the highest efficacy with the more effective therapy initially and then maintain it for a longer term with a safer medication [95,96,97].

In a retrospective review, 65 patients (80 studied eyes) underwent treatment involving pegaptanib + moxifloxacin administered every 6 weeks. The mean VA gain over 54 weeks was +9.2 letters for patients who did not receive previous medications, and +5.4 letters for those with prior treatment, such as PDT or triamcinolone acetonide [98]. Table 3 summarizes the efficacy results of pegaptanib among patients with AMD.

##### Diabetic Retinopathy (DR) and Diabetic Macular Edema (DME)

Diabetes mellitus (DM) poses a significant challenge in the developed world. One of its direct complications is diabetic retinopathy (DR), which can lead to vision loss through retinal ischemia, vitreous hemorrhage, retinal detachment, and diabetic macular edema (DME). DME involves the extravascular serum accumulation resulting from vascular leakage, leading to macular thickening. VEGF-165 plays an important role in the pathogenesis of DR and DME by causing vascular leakage. Notably, VEGF levels are often elevated in DME patients [99].

A randomized controlled double-masked dose-finding trial was conducted on patients with DME. The patients were divided into groups receiving 0.3 mg (n = 44), 1.0 mg (n = 44), or 3.0 mg (n = 42) of pegaptanib, or a sham injection (n = 42). The injections were administered at the beginning of the study, in weeks 6 and 12, and additional injections were given if necessary until week 30. The results were measured at week 36. The study found that 93%, 98%, 93%, and 90% of patients in the respective groups lost less than 3 lines, with 18%, 14%, 7%, and 7% gaining more than 2 lines. The mean central retinal thickness changes in the groups were −68.0, −22.7, −5.3, and +3.7 µm, respectively. Interestingly, similar to the V.I.S.I.O.N. study results, the 0.3 mg pegaptanib group performed the best and significantly better than the sham group. The study’s limitations include the small sample size and the moderate follow-up period [100]. Based on the data from this study, a retrospective, exploratory study was conducted with 16 subjects who had diabetic neovascularization at the baseline. 13 patients received pegaptanib (either dose) and 3 received sham injections. 62% of pegaptanib-treated patients showed regression, while none of the sham-treated patients did. However, after discontinuing the pegaptanib therapy, the progression of neovascularization resumed. These results suggest the potential role of pegaptanib in the treatment of DME [101]. However, the study faced criticism due to the notably small sample size, particularly the limited representation of 3 patients in the sham group, which does not allow for adequate statistical analysis. It was also highlighted that 90% of patients who showed regression were previously treated with panretinal photocoagulation [102].

In a retrospective study, the data of patients treated with pegaptanib alone (n = 13), together with macular laser photocoagulation (n = 12), or with laser alone (n = 15) were assessed. The mean VA change was −0.16 ± 0.15 in the pegaptanib group, −0.06 ± 0.14 in the combined treatment group, and 0.03 ± 0.14 in the laser group. The change in CMT was −146.77 ± 93.9 µm in the pegaptanib group, −71.67 ± 105 µm in the combination group, and −19.2 ± 54.2 µm in the laser group. Changes in VA were only significant in the pegaptanib group, while the reduction in CMT was significant in the pegaptanib and combined treatment groups. This suggests that pegaptanib is more effective for treating DME than macular laser photocoagulation [103]. A 64-year-old patient with DME who did not respond to grid laser photocoagulation was treated with pegaptanib. The patient received injections every 6 weeks for 6 months (5 injections) with a follow-up period of 42 months. CMT decreased from 511 µm to 376 µm by the 4th month and exhibited sustained stability, remaining within 10% deviation, during the subsequent follow-up period. Posterior hyaloid detachment was detected in the patient [104].

A 29-year-old female patient diagnosed with severe nonproliferative diabetic retinopathy who underwent panretinal photocoagulation and pars plana vitrectomy (PPV) was treated with a single intravitreal injection of pegaptanib. Subsequently, her VA improved from 30/200 to 80/200 in the third week, 120/200 in the 6th month, and 180/200 in the 15th month. Notably, complete regression of disk neovascularization was observed [105].

In a retrospective analysis, 63 eyes of 48 patients were reviewed with a minimum of 6 months of follow-up. The mean VA was 0.66 ± 0.37 (logMAR) at the baseline, 0.62 ± 0.38 after 6 weeks, and 0.53 ± 0.37 at the end of the follow-up. The mean CMT was 533.1 ± 123.1 µm at the baseline, 456.4 ± 123.9 µm at the 6th week, and 373.7 ± 135.8 µm at the end of the follow-up. The improvements in VA and the decrease in CMT were statistically significant [106].

In a prospective randomized controlled study, 20 patients with proliferative diabetic retinopathy (PDR) were randomized into 2 groups. One group received pegaptanib (0.3 mg every 6 weeks for 30 weeks), while the other received panretinal laser photo-coagulation (PRP). The pegaptanib-treated group showed an improvement of 5.8 letters in VA in week 3, 5.1 letters in week 6, and 5.8 letters in week 36. In the PRP group, the mean change in VA was −3.0, +3.0, and −6.0 letters at weeks 3, 6, and 36, respectively. However, the difference was not statistically significant. In the pegaptanib-treated group, the neovascularization regressed completely by week 12 and this improvement was maintained until week 36. In the PRP group, only 2 eyes showed complete regression and 2 showed partial regression at week 36. The mean CMT in the pegaptanib group changed from 201 µm to 178 µm in week 3, 175 µm in week 6, and 191 µm in week 36. In the PRP group, the baseline was 232 µm, with measurements of 229, 226, and 303 µm in weeks 3, 6, and 36, respectively. The differences in neovascularization regression and macular thickness observed were significant [107].

15 eyes of 14 patients with vitreous hemorrhage underwent treatment with pegaptanib, receiving 1–3 injections over a follow-up period of 6–24 months. All treated eyes showed no further vitreous hemorrhage for at least 4 weeks after pegaptanib treatment. This absence of vitreous hemorrhage was maintained in five eyes until the end of the study. Additionally, two other patients received further pegaptanib treatment, which helped them avoid vitrectomy throughout the study. If vitrectomy was necessary, it was found to be faster and easier to perform, as per the surgeon’s opinion. These findings suggest that pegaptanib may be used to prevent vitrectomy or at least make the procedure less challenging for diabetic retinopathy patients with vitreous hemorrhage. Furthermore, the patients’ VA improved, with the main gain in VA being 0.8 logMAR units (or 8 ETDRS lines) [108].

To examine the efficacy and safety of pegaptanib in patients with DME involving the center of the macula associated with vision loss not due to ischemia, a phase II/III randomized controlled trial was conducted. In the trial, patients were treated with pegaptanib (0.3 mg every 6 weeks for 1 year, 9 injections in total, n = 133) or received a sham injection (n = 127). Results showed that 36.8% of the pegaptanib-treated group gained at least 10 letters of VA at week 54, compared to 19.7% in the sham group. The mean VA increase in week 54 was 5.2 letters in the pegaptanib group and 1.2 letters in the sham group. Additionally, 31.7% of the pegaptanib group and 23.7% of the sham group showed at least a 25% decrease in central point thickness in week 54. A subgroup of 207 patients (107 in the pegaptanib group and 100 in the sham group) were followed up and assessed at week 102. The results showed that 38.3% of the pegaptanib-treated group gained at least 10 letters of VA in week 102, while in the sham group, the percentage was 30.0%. The mean VA increase was 6.1 and 1.33 letters in the 102nd week in the pegaptanib and sham groups, respectively. Furthermore, 40.4% of the pegaptanib group and 44.6% of the sham group showed at least a 25% reduction in thickness in week 102 [109]. The pegaptanib-treated group also experienced a greater improvement in VA-related quality of life [110]. As patients with macular ischemia were excluded from this Phase II/III trial, another trial was conducted specifically for subjects with ischemic DME. In this trial, pegaptanib was administered every 6 weeks for a total of 5 injections, and the final visit took place 6 weeks after the last injection. However, the study found no statistically significant changes in the area of the foveal avascular zone in the entire study population or in subgroups. Additionally, changes in central subfield thickness and VA were also not significant [111].

In a longitudinal interventional study, 30 patients with clinically significant edema were treated with pegaptanib 3 times at 6-week intervals, with a follow-up period of 48 weeks. The mean foveal thickness was 551.5 ± 129.8 μm at baseline, 246.4 ± 45.8 μm at 18 weeks, 269.4 ± 66.2 μm at 24 weeks, and 237.4 ± 41.1 μm at 48 weeks. VA also improved from 18.2 ± 8.5 letters (ETDRS chart) to 21.2 ± 8.7 letters at 18 weeks, and to 25.5 ± 8.4 letters at the final check. Additionally, an improvement in macular sensitivity and color discrimination was observed [112].

In a prospective, open-label case series, 6 patients with macular edema due to diabetic retinopathy were enrolled. The patients received 3 pegaptanib injections at 6-week intervals. After the third injection, a reduction in the blood flow velocity of the central retinal artery was observed. VA and macular thickness did not change significantly [113]. Table 4 summarizes the efficacy results of pegaptanib among patients with DR and DME.

##### Coats Disease

Coats disease is a rare disease of mostly unknown etiology, typically occurring in young males. The breakdown of the blood-retinal barrier plays a role in the pathogenesis, causing leakage that can result in retinal detachment [114,115]. A case was reported of a two-year-old boy with stage 4 coats disease. His VEGF level was 908 pg/mL, significantly higher than the normal level (100 pg/mL in adults). Treatment with pegaptanib resulted in an 80% reduction in exudation and decreased retinal detachment, with VEGF levels dropping to 167 pg/mL [114]. In a prospective, interventional case series involving 3 patients, one, a 16-year-old boy, was treated with pegaptanib, and the other two with different anti-VEGF therapies. After pegaptanib treatment, the boy’s VA stabilized, and macular edema and telangiectatic vessels decreased, along with a reduction in macular thickness 6 months after treatment [116].

##### Retinal Vein Occlusion

Retinal vein occlusion (RVO) is the obstruction of retinal outflow vessels. There are two types: central retinal vein occlusion (CRVO) where the main outflow vessel is obstructed, and branch retinal vein occlusion (BRVO) where a proximal branch of the central retinal vein is involved. RVO is associated with retinal ischemia, which in turn increases VEGF production, causing edema. Anti-VEGF agents, such as pegaptanib, have been tested for their effectiveness against RVO [117].

A dose-ranging phase II trial was carried out involving patients with CRVO. The subjects received pegaptanib at doses of 0.3 mg (n = 33) or 1.0 mg (n = 33), or sham injections (n = 32) every 6 weeks. At week 30, VA was measured. It was revealed that 36% of patients in the 0.3 mg group, 39% in the 1.0 mg group, and 28% in the sham group gained at least 15 letters, indicating a statistically nonsignificant difference. However, patients gained 7.1 and 9.9 letters on average in the respective pegaptanib groups, while in the shame group, patients lost 3.2 letters on average. Additionally, 9% and 6% of patients in the pegaptanib groups lost 15 or more letters, compared to 31% in the sham group. This suggests that although the primary endpoint did not show a significant difference, secondary analysis revealed a significant difference in the ratio of patients losing 15 or more letters in favor of the pegaptanib groups [118].

20 patients with BRVO were involved in a prospective randomized uncontrolled dose-finding study. Of these, 15 patients received 0.3 mg and 5 received 1.0 mg pegaptanib. The baseline VA was 56 ± 12 letters (or 20/80). At week 1, an improvement of 11 ± 7 letters was achieved, and by week 54, this improvement increased to 14 ± 13 letters. Center point thickness decreased from 489 ± 121 μm to 291 ± 76 μm in the first week and to 284 ± 152 μm in the 54th week, indicating rapid and sustained improvement. Notably, there were no significant differences between the two dose-based groups. However, one patient suffered retinal detachment [119]. A flaw in the study design was the inclusion of patients with BRVO for 1–6 months, as macular edema sometimes resolves without treatment during this time period. Thus, typically, 3 months are allowed for spontaneous recovery in the case of BRVO before initiating treatment. Additionally, some patients received grid laser treatment, making it difficult to differentiate the effect of pegaptanib treatment from that of other received treatments [120].

In a prospective, nonrandomized interventional study, 5 patients presenting with macular edema secondary to BRVO, refractory to bevacizumab or triamcinolone acetonide, were examined. They received two injections of pegaptanib administered every 5 weeks. After the first pegaptanib treatment, macular edema reduced from 418 ± 97.7 µm to 253 ± 97.25 µm within three weeks. VA improved at least 2 lines in all cases. These improvements observed remained stable for 3 months after the second treatment [121].

##### Choroidal Neovascularization in Different Diseases

A 36-year-old Asian female patient had choroidal neovascularization due to myopic degeneration. The patient’s VA declined despite being treated with PDT. However, after receiving 5 pegaptanib injections at 6-week intervals, her VA improved to 20/40 (baseline: counting fingers = CF) [122].

In a nonrandomized prospective clinical trial, 20 patients diagnosed with myopic choroidal neovascularization were administered pegaptanib injections at 6-week intervals. Over the course of 48 weeks, the mean VA increased from 25.5 ± 8.09 letters to 45.5 ± 8.16, and foveal thickness decreased from 271.3 ± 18.3 μm to 182.7 ± 8.5 μm. Notably, the macular sensitivity of the patients was also improved [123].

A 20-year-old patient with idiopathic choroidal neovascularization and 20/80 VA was initially treated with ranibizumab, resulting in an increase in VA to 20/20 −1. To maintain this result, pegaptanib was administered. Since the patient was a young female, pegaptanib was chosen over ranibizumab for the second treatment due to concerns about the potential impact of ranibizumab on the patient’s menstrual cycle, and the unclear teratogenic effects at that time. During a 4-month follow-up, no recurrence was detected [124].

Angioid streaks are irregular lines around the optic disk that extend radially outwards and are often associated with CNV. A 41-year-old patient with a VA of 45 ETDRS letters was treated with pegaptanib every 6 weeks, totaling 4 injections. By week 36, the VA had improved to 79 letters, intraretinal edema had resolved, and there was a significant improvement in pigment epithelial detachment [125]. five eyes of four patients with CNV secondary to angioid streaks were treated with pegaptanib and followed up for 15–24 months. VA improved in two eyes and stabilized in three eyes. Ultimately, only one patient showed minimal leakage [126].

A 14-month-old patient presenting with persistent fetal vasculature syndrome (PFVS), characterized by the lack of regression of fetal vasculature, received treatment with pegaptanib at a dosage of 0.3 mg, equivalent to the adult dose. 6 weeks later, the lesion size and leakage were reduced. By week 14, no leakage was detected and the lesion was involuted [127].

In a retrospective case series, the treatment of pediatric choroidal neovascular membranes (CNVMs) was documented. A male patient, aged 8, who had suffered blunt trauma to the left eye, was treated with pegaptanib. 6 months after the first injection, there was no change in VA from the original measurement of 20/200. Therefore, a second injection was administered. After 6 months, VA improved to 20/30 and remained stable for 14 months [128].

##### Macular Edemas

The effect of pegaptanib on macular edema of different causes was examined.

The case of a 59-year-old male patient with tamoxifen-induced macular edema was documented. Pegaptanib decreased macular thickness and leakage and improved visual acuity [129].

A prospective, nonrandomized interventional case series was reported involving four patients with refractory pseudophakic cystoid macular edema (CME), which is a common complication of cataract surgery. Within one month, the mean macular thickness decreased from 412 ± 20 μm to 239 ± 20 μm. Additionally, the mean VA improvement was 17 letters [130]. In another case study, a 33-year-old patient with CME secondary to pericentral retinitis pigmentosa (PRP) was initially treated with acetazolamide (500 mg daily) for one month without response. Subsequently, the patient received pegaptanib, and after one month, VA improved from 20/200 to 20/40. No recurrence of CME was observed after 4 months. As the patient continued the acetazolamide therapy, the individual effects of the two medications could not be distinguished [131].

Acute retinal necrosis syndrome is an infectious retinitis caused by herpes viruses. It can lead to retinal arterial obstruction, resulting in ischemia which in turn increases VEGF levels. A 35-year-old patient with bilateral acute retinal necrosis initially responded well to treatment (antiviral therapy, vitrectomy, and laser), but later developed severe cystoid macular edema in the right eye. Due to a family history of glaucoma and increased cataract risk, pegaptanib treatment was chosen instead of steroids to decrease intraocular inflammation. After 2 weeks of pegaptanib treatment, CFT decreased from 410 μm to 197 μm, and VA improved from 20/60 to 20/25 [132].

Irvine-Gass syndrome is a condition where macular edema develops after cataract surgery. A glaucomatous male with a VA of 20/125 was unsuccessfully treated with indomethacin. Given the elevated risk associated with steroid therapy in glaucoma patients, steroid treatment was declined, and pegaptanib was administered instead. After one week, the patient’s VA improved to 20/25, and near-complete resolution of macular edema was observed. These positive outcomes were sustained over 6 months [133].

##### Other Diseases

Von Hippel-Lindau (VHL) disease is an inherited tumorous condition, with retinal angioma being a common manifestation. In a pilot study, five VHL patients were treated with pegaptanib (a minimum of 6 injections at 6-week intervals). two patients successfully completed the one-year follow-up period, with one patient gaining 3 lines of VA, albeit without significant changes in leakage or tumor size. In the remaining three patients who did not complete the treatment regimen, the disease continued to progress. Moreover, one patient encountered retinal detachment [134].

A patient diagnosed with idiopathic parafoveal telangiectasis underwent treatment with pegaptanib. Following one month, the leakage, determined by fluorescein angiography, was reduced, but no changes in VA were observed. Two months later, although the leakage returned, VA remained stable. Another dose of pegaptanib was administered, which once again decreased leakage, but VA remained stable [135].

A patient presented with choroidal melanoma developed radiation retinopathy 14 months following episcleral plaque radiotherapy. This condition stems from radiation therapy-induced damage to blood vessels, causing hypoxia, which in turn induces an increase in VEGF level, thereby promoting neovascularization. The case was initially treated with laser photocoagulation, which proved ineffective, as disk neovascularization and retinal exudation were observed after 3 months, along with a decrease in the patient’s VA reaching 20/200. Pegaptanib was given as a treatment, and after one month, the patient’s VA improved to 20/40, the exudation disappeared, and disk neo-vascularization decreased [136].

In a retrospective case series, four patients (aged 6–23 years) with stage 3 or worse vascularly active familial exudative vitreoretinopathy (FEVR), a hereditary disease characterized by retinal neovascularization and subretinal exudation, underwent treatment with a single injection of pegaptanib. Before this treatment, the patients had not responded to photocoagulation, and cryotherapy and/or intravitreal steroids. 6 weeks after pegaptanib treatment, the leakage decreased and VA improved. However, two patients later developed vitreous hemorrhage, which was treated with pars plana vitrectomy, resulting in improved VA and reduced exudation [137].

A patient with idiopathic retinal vasculitis presented with persistent nonclearing vitreous hemorrhage and was treated with pegaptanib after initial treatments, such as photocoagulation, pars plana vitrectomy, cryotherapy, and prednisolone, all of which were ineffective. The patient’s VA improved from 6/36 to 6/18, and vascular leakage ceased 2 weeks after the pegaptanib injection. These positive outcomes remained stable for 9 months [138].

A retrospective review of 16 patients with retinal angiomatous proliferation (RAP) was reported. One patient discontinued the pegaptanib treatment due to a lack of improvement. For the remaining 15 patients, VA decreased from 45 ± 11 letters to 40 ± 14 letters in week 24, which was significant. 87% of the patients lost less than 15 letters. CFT did not change significantly until the 24th week. Leakage persisted in 13 patients. Although this disease is progressive, the potentially stabilizing effect of the treatment can still be beneficial [139].

Sturge-Weber syndrome (SWS) is a rare congenital disease including port-wine birth-mark (PWB) on the face, neurological disorders, and glaucoma [140]. A 13-year-old patient with SWS-related exudative retinal detachment, refractory to external beam radiation therapy, was treated with a single injection of pegaptanib. One week later, a 50% resolution of the detachment was observed, and after one month, a 100% resolution was achieved. These results remained stable for 6 months. However, despite this success, VA remained poor in the affected eye [141].

In a retrospective study, the efficacy of anti-VEGF agents against iris neovascularization was investigated. One studied eye received pegaptanib. The intraocular pressure decreased from 40 mmHg to 14 mmHg in the first month and to 11 mmHg in the 8th month. VA was 6/60 at the baseline, and there was no light perception at the final visit. However, vitrectomy surgery was performed during the study period [142].

In a pilot study, patients with chronic central serous chorioretinopathy (CCSCR) were treated with PDT and pegaptanib. Eight eyes of six patients received full-fluence PDT and anti-VEGF therapy (bevacizumab for four eyes and pegaptanib for four eyes), while the control group (10 patients) received only PDT. In the study, group, VA improved from 0.6 (or 20/80) to 0.2 (or 20/30), and CMT decreased from 288.4 ± 79.8 µm to 163.1 ± 25.9 µm. No leakage was seen in the eyes, and macular neurosensory retinal detachment (MNSRD) was resolved in 100% of the eyes. In the control group, VA was 0.7 (20/100) at baseline and 0.6 (20/80) at the 12th month. Macular thickness also decreased from 332.9 ± 85.6 µm to 213.1 ± 54.2 µm. 70% of patients showed leakage, and MNSRD was resolved in 70% of the eyes [143].

A prospective comparative study included 152 eyes of 76 patients with retinopathy of prematurity (ROP). Among them, 34 patients received pegaptanib + diode laser photocoagulation, while 42 received laser + cryotherapy. The study revealed a stable regression of stage 3+ ROP in 89.7% of the patients in the pegaptanib group compared to 60.8% in the other group [144].

82 patients with vascular active vitreoretinopathy (VAVR) were either given a single pegaptanib injection or a placebo. After 12 months, the pegaptanib group showed significantly lower leakage and inflammation. Additionally, VA improved from 0.3 to 0.11 in the pegaptanib group, and from 0.32 to 0.15 in the placebo group [145].

Although pegaptanib was re-evaluated to be used as a maintenance therapy after ranibizumab was approved for treating wet AMD, its story did not end there. Pegaptanib is associated with fewer side effects and better systemic tolerability (see more below), making it a more suitable option in certain clinical scenarios, such as for patients with DME who require long-term treatment and are at high risk for cardio- and cerebrovascular events. This is important since plasma VEGF plays a fundamental role in protecting the blood vessels and maintaining the anti-thrombotic and anti-inflammatory properties of the endothelium, and prolonged suppression from anti-VEGF injections may have undesired systemic effects [146,147]. Taking this into consideration, some authors have suggested using pegaptanib as an initial treatment for DME, to be replaced by a pan-VEGF blocker if a positive outcome is not achieved. Additionally, despite the efficacy of ranibizumab in wet AMD, a significant number of patients still experience poor outcomes. In some instances, pegaptanib has proven effective. One possible explanation for this is that anti-VEGF antibodies bind to both the pro-angiogenic VEGF-165 and the anti-angiogenic VEGF-165b (see more below) with equal affinity [148]. Research conducted in cancer models indicates that VEGF-165b can significantly undermine the efficacy of bevacizumab, suggesting that patients with elevated levels of VEGF-165b may be less responsive to bevacizumab and other pan-VEGF agents [47]. Given the ongoing interest in pegaptanib, it is possible that we may see it regain wider application in the near future.

#### 2.3.3. Safety and Adverse Events

##### General Safety

Some aspects of safety were briefly mentioned above. Initially, there were serious concerns about the potential systemic effects of anti-VEGF treatment [60], but this turned out to be a false alarm. The main adverse events found in the V.I.S.I.O.N. trial were local reactions attributed mostly to the administration method rather than the active ingredient itself. The common adverse events, such as eye pain, vitreous floaters, cataract, vitreous opacities, corneal edema, and anterior chamber inflammation were mild to moderate and transient. Serious adverse events were rare; for example, ophthalmitis occurred in 1.3%, and retinal detachment in 0.7% of the pegaptanib-treated group [61]. It was also shown that pegaptanib can be an effective and safe therapeutic option for patients with a high risk of arterial thromboembolic events [88].

Parallel to the 2-year efficacy study [68], the 2-year safety results were also published [149]. To summarize the results: in the first year, 892 patients received at least 1 dose of pegaptanib (0.3, 1.0, or 3.0 mg) and 298 received a sham treatment (1190 subjects in total). A total of 7545 injections of pegaptanib were administered during this period. Ocular adverse events occurred in 92% of the patients who received pegaptanib (19% of them were serious) and in 87% of the patients who received the sham treatment (15% were serious). The most common events in the pegaptanib groups were eye pain (34%), vitreous floaters (33%), punctate keratitis (32%), and increased intraocular pressure (IOP) (20%). Among the pegaptanib-treated patients, twelve developed endophthalmites, one suffered severe vision loss (at least 30 letters lost), and eight lost 10 or fewer letters. In 75% of endophthalmitis cases, a violation of the injection preparation protocol was reported, which may have contributed to this adverse event. Additionally, six retinal detachments were reported, with two of them possibly being secondary to the underlying disease. Five traumatic cataracts were reported, all of which were iatrogenic (caused by the injection). Four patients experienced transiently decreased central retinal artery perfusion after pegaptanib injection, along with increased IOP. Retinal vein occlusion was not observed. The incidence of vitreous hemorrhage was sixteen cases/7545 injections. A mild increase (2–4 mmHg) in IOP was noted post-injection, which returned to normal within a week. No local vasculature toxicity was evident, and systemic toxicity was not detected. The incidence of cardiac events was higher in the sham group compared to the pegaptanib group (5% vs. 2%). Both groups had a 2% mortality rate, which is to be expected, considering the advanced age of the participants. Pegaptanib was not linked to potential systemic anti-VEGF effect-related events, such as thromboembolic events. In the second year of the study, 425 patients continued with the same treatment as in the first year (cohort 1), 439 patients were re-randomized to discontinue the treatment (cohort 2), while 160 patients who had previously received a sham treatment were re-randomized to be treated with pegaptanib (cohort 3). A total of 2663 pegaptanib injections were administered during this period. In cohort 1, the most common ocular events were eye pain (25%), increased IOP (24%), punctate keratitis (23%), and vitreous floaters (22%). Most of these events were transient and mild or moderate in nature. It was also noted that the incidence of these events was higher in the study eye of the sham group than in the fellow eye of the pegaptanib group, suggesting that the injection procedure itself, rather than the active ingredient, was the main cause of these events. Four cases of retinal detachment were reported. The mean IOP increase after injection was 2–6 mmHg. No unexpected vascular abnormalities were detected, and there were no signs of systemic toxicity. Overall, the safety results were similar to those of the first year. In cohort 2, no safety issues were reported, although one case of retinal detachment was observed. In cohort 3, four cases of endophthalmitis, two cases of retinal detachment, and one case of iatrogenic traumatic cataract were documented. The safety outcomes were similar to those of the first year. Across all three cohorts, the overall incidence of endophthalmitis in the second year was 0.1% per injection, traumatic cataract was 0.02% per injection, retinal detachment was 0.17% per injection, and vitreous hemorrhage was 0.27% per injection. None of the four endophthalmitis cases suffered severe vision loss (more than 30 letters). Overall, the 2-year safety profile of pegaptanib proved to be favorable [119]. It is important to note that individuals with a history of severe cardiac disease, myocardial infarction, or unstable angina were excluded from the study, impacting the systemic safety findings [150].

After the second year, patients who received 0.3 mg or 1.0 mg of pegaptanib treatment in the first or second year continued with the same dosage, while those who received 3.0 mg or did not receive pegaptanib in the first 2 years were re-randomized to receive either 0.3 mg or 1.0 mg of pegaptanib for the third year. The results in the third year were similar to those in the previous years. Altogether, 422 subjects were involved, and a total of 3227 injections were administered in year 3. The primary safety population consisted of the patients who received pegaptanib in the first 2 years and received at least one dose of pegaptanib in the third year, which included 161 patients. 71% of patients experienced ocular adverse effects. The most common were punctate keratitis (25%), increased IOP (20%), eye pain (17%), and cataract (14%). Additionally, 17% experienced serious ocular or non-ocular adverse events. The most common non-ocular adverse events were infections (18%), respiratory, thoracic, and mediastinal disorders (15%), and gastrointestinal disorders (14%). No thromboembolic cerebrovascular events or serious non-ocular hemorrhagic events were reported. Myocardial infarction was reported by 2% of the subjects, and angina by 1%. Among the 422 subjects, the incidence of endophthalmitis was 0.47% and rhegmatogenous retinal detachment was 0.24%. No traumatic cataract was reported. IOP changes were generally similar to those in the previous years. The most frequent serious adverse events were neoplasms and cardiac disorders (3%), GI disorders (2%), and vascular disorders (2%), which are not unexpected in an elderly population. There were six deaths, but none of them were attributed to pegaptanib. No meaningful change in blood pressure or laboratory parameters was detected. These results suggest a favorable safety profile and are consistent with previous findings. There was a decrease in the incidence of endophthalmitis, likely due to the change in the administration technique [151]. It is worth noting that these results are comparable with the general rate of myocardial infarction (2.2%) and cerebrovascular events (3.8%) observed in the neovascular AMD population [152].

147 patients with AMD-related CNV were involved in two studies: an open-label cohort and a randomized, double-masked, uncontrolled clinical trial. In the open-label cohort, 37 patients received 3 mg pegaptanib every 6 weeks for 54 weeks. In the double-masked randomized cohort, 54 patients received 1.0 mg, and 56 patients received 3.0 mg pegaptanib. Throughout the studies, liver and renal function, hematology, and electrolyte parameters were monitored, and no clinically significant changes were observed. Blood pressure did not show any meaningful changes that could indicate a systemic anti-VEGF effect. No anti-pegaptanib antibodies were detected in any patients’ serum, although this result is uncertain due to the lack of a positive control. 94% of patients experienced ocular adverse events, mostly mild to moderate. One serious ocular adverse effect (retinal hemorrhage) was reported but was not considered to be related to pegaptanib. Endophthalmitis, retinal detachment, or traumatic cataract were not reported. The most common ocular adverse events included eye pain (47%), vitreous floaters (43%), punctate keratitis (41%), visual disturbance (25%), eye irritation (22%), and increased IOP (21%). Mild AC (anterior chamber) inflammation was reported by 7% of patients. The mean change in IOP 30 min after injection was 3–8 Hgmm and was transient. No systemic safety concerns were reported. It is important to note that the doses used were higher than the effective dose determined based on the phase III results. Despite this, the efficacy of pegaptanib did not increase above the 0.3 mg dose, while some adverse effects did. For instance, the increase in IOP was greater in the 3.0 mg group than in the 1.0 mg group [153].

The safety profile of pegaptanib for treating patients with DM was evaluated in a pooled, retrospective analysis of data from 9 trials. The analysis included a total of 1586 patients, with 165 having a history of DM and 1421 without. The study found that treatment-emergent adverse events, such as eye disorders, were reported in 71% of the DM group and 77% of the non-DM group. Additionally, 17% of the DM group and 21% of the non-DM group reported prespecified ocular adverse events. Thromboembolic adverse events were reported by 6.1% of the DM group and 4.2% of the non-DM group. In terms of mortality, there were 6 deaths (3.6%) in the DM group and 28 deaths (2%) in the non-DM group. One limitation of this study is the imbalance in the size of the two groups. Overall, no notable differences between the two groups were identified, suggesting that DM does not pose an increased risk for pegaptanib treatment [154].

A total of 16 DME patients were treated with pegaptanib at a dosage of 0.3 mg every 6 weeks or less frequently, with a median interval of 6.5 weeks between injections, over a period of 1 year. Among the patients, 21.7% reported ocular adverse events, none of which were severe. Additionally, 17.4% reported non-ocular adverse events, with two severe cases not related to the treatment. One patient reported a moderate hypersensitivity (skin reaction), which was unrelated to the treatment [155].

The charts of patients receiving anti-VEGF treatments were reviewed. Out of 29 patients treated with pegaptanib, six patients (20.7%) experienced side effects. Among those, one experienced high blood pressure (>200 mmHg), four had a transient increase in IOP, and one had a sustained elevation in IOP [156].

A population-based study of 504 patients with neovascular AMD found no association between anti-VEGF therapy, including pegaptanib (n = 10), and stroke, myocardial infarction, or death [157].

After discussing the overall safety outcomes of pegaptanib, we briefly address a few specific issues.

##### Endophtalmitis

Endophthalmitis was one of the first complications identified. In the V.I.S.I.O.N. study, 1% of patients experienced endophthalmitis, and among those, one subject lost 30 or more letters of VA. Two-thirds of the endophthalmitis cases had positive cultures, with coagulase-negative *S. epidermidis* being the most common pathogen. Additionally, 67% of the cases were linked to violations of the injection protocol. The incidence of endophthalmitis per injection decreased over time, showing rates of 0.16% in the first year, 0.1% in the second year, and 0.06% in the third year. This decline is likely due to the implication of stricter administration protocols [151].

In a retrospective interventional case series, a total of 10254 intravitreal injections of anti-VEGF agents (406 pegaptanib) were administered. Only three cases of endophthalmitis were reported, and none occurred among pegaptanib-receiving patients [158]. In another study, similar findings were observed. In a cohort of 30736 anti-VEGF injections (128 pegaptanib), 15 cases of endophthalmitis were detected, with none following pegaptanib injections [159]. A review of 3875 anti-VEGF injections revealed an endophthalmitis rate of 0.08% per injection (Pegaptanib was not discussed separately in this study) [160]. In a Japanese study, five patients developed endophthalmitis out of 5236 administered anti-VEGF injections. This included 200 injections of pegaptanib; however, none of the cases occurred in patients treated with pegaptanib [161].

Twelve cases of endophthalmitis were reported following 60322 anti-VEGF injections (2015 pegaptanib injections), but none occurred among patients who received pegaptanib [162]. Over a span of ten years, 33580 intravitreal injections were reviewed (with 984 being pegaptanib), resulting in 109 cases of endophthalmitis, 21 of which were post-injection, and none after pegaptanib injections [163]. In another study involving 608 patients who received anti-VEGF injections, 428 received pegaptanib, and no cases of endophthalmitis were reported [164]. Over a five-year period, 15895 intravitreal injections were reviewed (including 121 pegaptanib injections). Nine cases of endophthalmitis were identified, none of which received pegaptanib [165]. The limited number of pegaptanib injections in most of these studies presents an important limitation; however, the general trend indicates that the use of anti-VEGF therapy does not carry a high risk for endophthalmitis.

There was a report of an 84-year-old woman who experienced acute endophthalmitis following pegaptanib treatments, necessitating intravitreal antibiotic injections (vancomycin and ceftazidime) and vitrectomy [166]. In another case, an 80-year-old woman developed endophthalmitis after a pegaptanib injection and was treated with a vitreous tap and antibiotic injections. A vitrectomy was also needed [167].

It has been shown that the risk of endophthalmitis can be further reduced by following an adequate protocol. In a study where povidone-iodine irrigation was used pre- and post-injection among 15,144 administered injections (548 of which were pegaptanib), no cases of endophthalmitis were detected [168].

##### Increased Intraocular Pressure (IOP)

In the clinical trials previously presented, an increase in IOP was a common, but mild and transient adverse effect [150,151,152,153,156]. In a post hoc analysis of the V.I.S.I.O.N. study, the data of 114 patients in the pegaptanib (0.3 mg) group and 107 subjects in the sham group were reported. The ratio of participants with sustained IOP increase (at least a 22 mmHg increase from the pre-injection IOP one week after the injection or at any unscheduled measurement) was 24.6% in the pegaptanib group and 21.5% in the sham group over two years. No patients exhibited >30 Hgmm sustained IOP increase in either group [169].

In a review of 79 patients’ charts (which included 122 pegaptanib injections), the baseline IOP was 15.73 ± 0.41 mmHg. 30 min after pegaptanib injection, the mean IOP rose to 24.47 ± 6.29 mmHg, indicating a change of 8.74 ± 7.23 mmHg. By days 5 and 7, the IOP values normalized. Although 13% of eyes experienced an IOP increase >30 Hgmm, these also normalized by days 5 and 7. None of the patients required IOP-decreasing medication or paracentesis [170].

A total of 212 intravitreal injections were administered (including 94 pegaptanib injections across 74 patients). 36% of the pegaptanib-treated eyes showed >10 Hgmm increase in IOP within 30 min after injection. Once again, none of the patients required IOP-lowering drops [171].

In a retrospective interventional case series, the effect of anti-VEGF agent injections on the IOP was examined. The charts of 112 patients (120 eyes with 213 injections, including 29 pegaptanib injections) were reviewed. The mean IOP at the baseline was 14 mmHg (ranging from 7 to 22 mmHg), which increased to 44 mmHg (ranging from 4 to 87 mmHg) immediately after the injection. In 100% of cases, IOP was reduced to less than 30 mmHg within 30 min, demonstrating the transient nature of this change [172].

In a retrospective review, 1555 eyes of 127 patients received IV injections of the three anti-VEGF agents (bevacizumab, ranibizumab, and/or pegaptanib). Among them, 9.4% showed >25 Hgmm increase in IOP post-injection, and 5.5% showed sustained IOP elevation [173].

A retrospective study reviewed 75 pegaptanib-treated eyes in patients with and without glaucoma. The baseline pre-injection IOP was 14 ± 3 mmHg. One minute after the injection, the IOP rose to 38 ± 14 mmHg. 10 min post-injection, the IOP was 34 ± 9 mmHg. At 11–20 min, it decreased to 26 ± 10 mmHg, and at 21–30 min, it was 24 ± 11 mmHg. By 31–50 min, the IOP was 22 ± 3 mmHg. For most patients, the IOP returned to normal after 30 min, although for some, it took up to an hour. There was no significant difference in IOP responses between patients with or without glaucoma. Importantly, most patients had received IOP-lowering medication prior to treatment [174].

In a prospective study, 41 patients were randomized into two groups: those receiving anterior chamber paracentesis (Group A, 20 patients) and those not receiving it (Group B, 21 patients) before intravitreal pegaptanib injection. In Group A, the base-line IOP was 15.6 ± 4.3 mmHg, becoming 15.3 ± 7.5 mmHg 2 min after injection, and then 17.3 ± 4.5 mmHg at 30 min, with a final reading of 12.9 ± 3.7 mmHg one week later. In Group B, the values were 14.6 ± 3.4 mmHg (baseline), 47.1 ± 24.1 mmHg (2 min), 17.6 ± 5.0 mmHg (30 min), and 12.3 ± 2.5 mmHg (one week later). These results suggest that prophylactic anterior chamber paracentesis can prevent the transient increase in IOP after pegaptanib injection [175]. Another study found that IOP-lowering medications (timolol maleate, brimonidine tartrate, apraclonidine hydrochloride, dorzolamide hydrochloride, and brinzolamide) administered before the injection are not able to prevent IOP spikes [176].

##### Retinal Pigment Epithelial Tear

Retinal pigment epithelial (RPE) tear was not detected in the phase III trial; it was first reported in 2006.

Two patients developed RPE tear 1 and 8 weeks after pegaptanib injection. One possible mechanism for this occurrence is the vasoconstriction of the choroidal vessels caused by anti-VEGF therapy. However, since RPE tear can also arise as a complication of AMD or PDT, establishing a direct causal relationship cannot be determined based on these data. In the same year, another study reported two similar cases where patients exhibited RPE tear during a 6-week follow-up after pegaptanib injections [177,178]. Notably, the patients in both studies had pigment epithelial detachment (PED).

The charts of six AMD patients with PED who experienced RPE tear after pegaptanib treatment were reviewed. Four of these patients developed the tear within 8 weeks following their first injection, while the others experienced this after their second injection. The time interval between the last injection and the diagnosis of RPE tear ranged from 2 to 8 weeks. The incidence of RPE tears among pegaptanib-treated patients with PED was calculated to be 27%, compared to a spontaneous incidence of 10%. However, the small sample size limits the reliability of these results [179]. The time patterns in these studies suggest the causality between the pegaptanib injection and RPE tears.

A review also indicates a possible link between anti-VEGF agents and RPE tears [180]. Other studies found the RPE tear rates for eyes with vascularized PED after anti-VEGF therapy to be 17% [181], or 12–15% [182]. Furthermore, findings suggest that the larger basal diameters and vertical heights of PEDs act as risk factors for RPE tears [181,182].

##### Silicon Oil Droplets

Two cases were reported involving patients with presumably silicon oil droplets in their eyes following multiple intravitreal injections of pegaptanib. The occurrence of these droplets seemed to increase with the number of injections. Notably, similar droplets were seen in a third patient after receiving other intraocular injections, suggesting that the cause may not specifically be pegaptanib itself, but rather the lubricant used in the syringes [183].

In an observational case series that reviewed 1529 intravitreal injections, silicon oil droplets were detected in 15 eyes. Interestingly, the presence of these droplets did not affect the VA of the patients, and no intervention was required [184].

Among 22 patients who received intravitreal pegaptanib injections, silicon droplets were identified in three eyes. Mass spectrometry revealed the presence of silicon in both used and unused syringes, as well as in the pegaptanib solution. This further supports the assumption that the syringe lubricant is the source of these droplets [185].

##### Other Studies on Potential Side Effects and Drug Interactions

Among the previous studies, one hypersensitivity reaction was reported, which was assumed to be unrelated to pegaptanib. Additionally, no anti-pegaptanib antibodies were detected [153,155]. However, in 2007, two cases were documented. One patient experienced a prolonged anaphylactic reaction after receiving the first dose of pegaptanib, while the other developed a urticarial rash that subsided after discontinuing pegaptanib therapy [186].

The safety of pegaptanib in patients undergoing warfarin (an anticoagulant) therapy was evaluated through a retrospective chart review. This study included data from 31 patients, accounting for 32 eyes and a total of 102 pegaptanib injections. Among these patients, only one (who was also treated with acetylsalicylic acid) experienced acute submacular hemorrhage 35 days after the third pegaptanib injection. No other hemorrhagic adverse events were reported. These findings suggest that pegaptanib can be safely used in patients receiving warfarin; however, the study is limited by its small sample size and lack of control [187].

In another case, a patient with chronic serous drusenoid PED was treated with pegaptanib. One month post-treatment, foveal geographic atrophy with more than 175 μm diameter was developed, potentially due to decreased blood flow resulting from the anti-VEGF effect [188].

Additionally, two cases of patients with proliferative diabetic retinopathy were reported. In these instances, the previously stable tractional retinal detachment progressed after 3 and 5 weeks of pegaptanib injections [189]. Two cases of tractional retinal detachment were also noted during the treatment of patients with vascularly active FEVR [137].

Another case involved a 93-year-old woman who developed crystalline keratopathy 7 months after beginning pegaptanib treatment. The keratopathy healed spontaneously 4 months after discontinuing pegaptanib [190].

A 66-year-old patient with DME developed a lamellar macular hole one month after receiving a pegaptanib injection. It was unclear whether this event was caused by the injection or by pegaptanib itself [191].

A study involving 23 eyes from 21 patients examined the effect of anti-VEGF agents on the vertical cup-to-disk (C/D) ratio in the optic nerve. Of these eyes, 21 received pegaptanib, while two were treated with both pegaptanib and ranibizumab. The results indicated no significant change in the C/D ratio [192].

Additionally, it was found that long-term anti-VEGF therapy does not lead to an increase in retinal nerve fiber layer thickness [193].

A review of individual case safety reports for anti-VEGF medications in the VigiBase, covering the years 2010 to 2016, sought to evaluate any potential link between anti-VEGF treatment and dementia or Parkinson-like events. The review revealed no concerns regarding pegaptanib, as no cases were reported [194].

#### 2.3.4. Cost-Effectiveness

In addition to efficacy and safety, there has been a growing emphasis on the cost-effectiveness of new medicines, especially modern treatments, which are often more expensive to produce than traditional small-molecule drugs.

The cost of pegaptanib treatment in 2005 was 995 US$ per injection, meaning an annual cost of 8600 $ for one patient (considering 6-week intervals between injections) [195].

According to a 2007 article, the cost to the National Health Service (NHS) of the United Kingdom for a single pegaptanib injection was 514 £, translating to 4630 £ per year [196].

A British study evaluated the cost-effectiveness of pegaptanib compared to best supportive care (BSC) using a modeled population. In this analysis, pegaptanib was likely cost-effective, with an 8023 £ incremental cost/QALY. The estimated mean total lifetime cost/patient was 2383 £ higher for the pegaptanib-treated group than for the BSC group. However, the pegaptanib group experienced an average increase of 0.884 in vision years and a mean QALY increase of 0.297 compared to the BSC group. Additionally, cost-effectiveness could be improved by pre-treatment and proper discontinuation for non-responding patients [197].

A Canadian study examined the cost-effectiveness of pegaptanib compared to PDT with verteporfin and standard care, using a Markov model. Pegaptanib was found to be cost-effective compared to the other two groups. The incremental cost/QALY versus standard care was estimated at 59,039 CAD, and 49,052 CAD versus PDT. Additionally, the incremental cost/vision year gained was estimated to be 21,559 CAD compared to standard care, and 20401 CAD versus PDT [198].

The cost of a saved line of vision using pegaptanib was estimated at 12482 US dollars, with 17% allocated to professional fees and 70% to pharmaceutical costs [199].

An Indian study reported that one dose of pegaptanib is less expensive than one dose of PDT (45,000 INR vs. 65,000 INR). However, the expected number of treatments is lower for PDT (3 treatments) than for Macugen (20 treatments every 6 weeks). This results in a total cost of 195,000 INR for PDT and 900,000 INR for pegaptanib [200].

The cost-effectiveness of laser photocoagulation, PDT, and pegaptanib were compared. The QALY gains during a 12-year term were 0.301 for laser photocoagulation (4.4% improvement in quality of life), 0.438 for pegaptanib (a 5.9% improvement), and 0.585 for PDT (an 8.1% improvement). The cost/QALY values were 6684 $ for laser photo-coagulation, 59,787 $ for pegaptanib, and 27,945 $ for PDT [201].

A 2008 analysis suggests that the cost-effectiveness of pegaptanib is more favorable in patients with early-stage disease, with a total treatment cost estimated at 66,638 $ for the early-disease group and 96,771 $ for the late-stage group [202].

An economic evaluation published by NIHR estimated the cost of one year of treatment with pegaptanib at 4626 £, with additional expenses of 2614 £ for non-drug costs and another 1200–2100 £ for managing injection-related adverse events. Compared to usual care, the pegaptanib incremental cost-effectiveness ratio (ICER) was 163,603 £ in the 2-year model. Most costs are incurred in the first two years, and since QALY gains are low during this period, the ICER becomes more favorable in the long term, estimated to be 30,986 £ in the 10-year model [203].

A 2010 analysis found that over two years, PDT is more effective and less expensive for treating predominantly or minimally classic lesions in AMD [204].

#### 2.3.5. Comparing with Antibodies

Similarly to the frequent comparisons between antibodies and aptamers, pegaptanib faced competition from VEGF-targeting antibodies almost from the outset. Bevacizumab (AvastinTM) is a humanized monoclonal antibody targeting all soluble, circulating VEGF-A isoforms. Bevacizumab was originally approved in the US (2004) and in the EU (2005) as an anticancer agent, but subsequently found off-label application in ophthalmology [205]. Ranibizumab (LucentisTM) is an antibody fragment containing the modified antigen-binding part of bevacizumab. The smaller size grants better retinal penetration and more favorable pharmacokinetics [206].

##### Efficacy

Efficacy is where antibodies excel. This is likely due to bevacizumab and ranibizumab being non-selective anti-VEGF agents that target all VEGF isoforms, resulting in a greater biological effect. This is not just a hypothetical advantage in terms of anatomical or laboratory values. From the patient’s perspective, MABs are able to restore some of the lost vision, whereas pegaptanib predominantly attenuates disease progression or stabilizes visual acuity [207].

In the ANCHOR study, patients were randomly assigned to receive verteporfin PDT (n = 143), 0.3 mg ranibizumab (n = 140), or 0.5 mg ranibizumab (n = 140), with a 1-year follow-up period. The study found that 94% and 96% of patients in the 0.3 mg and 0.5 mg ranibizumab groups, respectively, lost < 15 letters compared to 64% in the verteporfin group. Furthermore, 5.7% and 40.3% of patients in the respective ranibizumab groups gained more than 15 letters, whereas 5.6% in the verteporfin group exhibited similar improvement [208].

In the MARINA study, patients were administered either ranibizumab (0.3 mg, n = 238 or 0.5 mg, n = 240), or sham injections (n = 238), with a 2-year follow-up. At the end of the first year, 94.5% of patients in the 0.3 mg group and 94.6% in the 0.5 mg group lost < 15 letters, compared to 62.2% in the sham group. By the end of the second year, these ratios were 92%, 90%, and 53% for the respective groups. Approximately 25% and 33% of individuals in the ranibizumab-treated groups gained <15 letters, this percentage was 5% in the sham group [209].

In the FOCUS study, patients received PDT verteporfin (n = 56) or ranibizumab + PDT verteporfin (n = 106). After 12 months, 90% of ranibizumab-treated patients lost < 15 letters, while 68% in the PDT alone group lost < 15 letters. In the combined therapy group, 24% gained <15 letters, compared to 5.4% of the PDT alone group [210]. At the end of the second year, 88% of ranibizumab-treated patients lost < 15 letters vs. 75% in the PDT alone group. 25% in the ranibizumab group gained <15 letters, compared to 7% in the PDT alone group [211,212].

In the first year of the V.I.S.I.O.N. study, 70%, 71%, and 65% of patients in the three respective pegaptanib groups lost < 15 letters, compared to 55% in the sham group. Additionally, 6%, 7%, and 4% of patients in the pegaptanib groups gained <15 letters, compared to 2% in the sham group [61]. These results indicate that ranibizumab is superior to pegaptanib in terms of the primary endpoint, with an even greater advantage noted in the secondary endpoint. However, it is important to consider that the ranibizumab RCTs involved fewer patients than the V.I.S.I.O.N. study, and direct comparisons cannot be made due to differences in the patient population’s lesion types [213].

Regarding bevacizumab, two small uncontrolled studies were published with positive results for its use in AMD [214,215]. The ABC trial was the first controlled randomized trial to explore the efficacy of bevacizumab in AMD. Patients were randomized to receive either bevacizumab (n = 65) or standard care (n = 66), with standard care including PDT, pegaptanib, or sham treatment. In the bevacizumab group, 32% of patients gained >15 letters of VA, compared to just 3% in the standard treatment group. Furthermore, 91% of patients in the bevacizumab group lost < 15 letters, while 67% in the standard treatment group experienced the same [216]. A 2012 analysis of OCT results from the ABC trial concluded that reductions in retinal edema and subretinal fluid were significantly greater in the bevacizumab group than in those treated with pegaptanib. Additionally, in the pegaptanib group, the reduction in subretinal tissue was not maintained [217].

There have also been studies comparing pegaptanib with antibodies directly. A retrospective case series of 53 patients was reported, with 18 receiving pegaptanib and 35 receiving bevacizumab. It was found that the mean total retinal volume reduction was higher in patients treated with bevacizumab [218].

In a prospective, comparative, non-randomized study focusing on patients with PED, it was observed that those treated with pegaptanib had a slower but more maintained improvement in VA and foveal thickness. Conversely, the bevacizumab-treated group experienced a more rapid improvement, but their results gradually returned to baseline levels. Altogether, the six-month results were more favorable for the pegaptanib group. However, this study had some serious limitations, including the small sample size (with only 7 patients receiving pegaptanib and 8 receiving bevacizumab), baseline differences between the groups, the lack of randomization, and the short follow-up duration [219]. Another study involving 328 patients (86 treated with bevacizumab, 128 with ranibizumab, and 60 with pegaptanib) found that ranibizumab and bevacizumab were significantly superior for treating PED in AMD [220].

The effects of pegaptanib and ranibizumab on neovascular AMD with small lesion sizes were compared in a retrospective study involving 81 eyes from 78 patients. VA was measured at months 1, 3, 6, and 12. The results showed no significant difference between the two treatment groups at any point during the study, indicating that pegaptanib can be considered equivalent to ranibizumab for treating wet AMD with small lesions [221].

In their 2013 meta-analysis, Ollendorf et al. found no statistically significant difference in the efficacy of anti-VEGF agents for the treatment of DME when examining changes in VA [222].

Despite pegaptanib’s seemingly lower efficacy, it can still be beneficial for persistent cases. In one study, 50 patients who were found to be resistant to ranibizumab or a combination of ranibizumab and PDT were treated with pegaptanib for 12 months. The VA was stable in 80% of the patients and improved in 18% of them. The mean VA (logMAR) was 0.53 ± 0.44 initially, 0.63 ± 0.41 before pegaptanib treatment, and 0.56 ± 0.42 at month 12. The CRT was 446.9 ± 150.6 μm initially, 414.5 ± 146.7 μm before pegaptanib treatment, and reduced to 318.7 ± 100.0 μm by the 12th month [223]. Table 5 provides a summary of the efficacy results of pegaptanib in comparison to anti-VEGF antibodies.

##### Safety

The safety concerns related to anti-VEGF therapy were particularly pronounced for ranibizumab and bevacizumab in comparison to pegaptanib, owing to the nonselective nature of these agents in targeting VEGF. For example, given the role of VEGF-A as a survival factor for the retinal neurons, the general anti-VEGF effect can lead to a decrease in neuron viability in the long term [224]. In ranibizumab clinical trials, a higher incidence of thromboembolic events was reported in the ranibizumab-treated groups, although the difference was not significant. Furthermore, there was a significant increase in the number of non-ocular hemorrhagic events in the ranibizumab group. Ranibizumab was also associated with a significant risk of stroke, but not myocardial infarction or vascular mortality [212].

Data from the Centers for Medicare and Medicaid Services were analyzed involving 94686 beneficiaries who were treated with anti-VEGF agents. The study found no increased risk of mortality, myocardial infarction, bleeding, or stroke for patients treated with bevacizumab or ranibizumab compared to those treated with PDT or pegaptanib [225].

An analysis of WHO-VigiBase data between 2002 and 2012 found a link between ranibizumab and cardiovascular adverse events and between bevacizumab and endophthalmitis and uveitis. For pegaptanib, no relevant safety concerns were identified [226].

A 2016 systematic review stated that there is no sufficient evidence in the literature to estimate the cardiovascular safety of bevacizumab in AMD [227].

A case report suggested that pegaptanib could serve as an alternative treatment for patients with hypersensitivity to ranibizumab or bevacizumab [228].

##### Cost-Effectiveness

In a Spanish study, Hernández-Pastor et al. analyzed treatment costs and health outcomes (Quality-adjusted life years—QALYs), to compare the cost-effectiveness of ranibizumab and pegaptanib. They found that using ranibizumab to treat AMD patients costs +71,206 € more than pegaptanib, but provides +2.437 QALYs, resulting in a cost of 29,224 €/QALY. This cost can be reduced to 4623 €/QALY with a modified administration. The study concluded that ranibizumab is a cost-effective option compared to pegaptanib [229]. However, the study was criticized for not taking into account all indirect costs [230].

However, a Canadian study found that the cost/+QALY of ranibizumab is greater than 50,000 $, making it unacceptably more expensive [231].

A Greek study found that for the treatment of predominantly classic lesions, ranibizumab is both cheaper and more effective. However, for minimally classic and occult lesions, ranibizumab is more effective but also more expensive, with an incremental cost-effectiveness ratio of 13,112 €/QALY and 28,561 €/QALY, respectively [232].

An analysis of Czech data found that ranibizumab therapy is less expensive (by 1220.16 €/year) while equally effective [233].

#### 2.3.6. Other Results

##### Efficacy and Safety in Nonclinical Trials

In a randomized controlled study, the researchers assessed the effects of subconjunctivally injected anti-VEGF agents on the inhibition of corneal neovascularization in an experimental rat model. Pegaptanib showed significantly fewer neovascularized corneal areas and fewer blood vessels, with no corneal epitheliopathy, compared to the control group. There was no significant difference between the effect of ranibizumab and pegaptanib, and bevacizumab was shown to be the most effective agent [234]. Another study revealed that pegaptanib significantly reduced the percentage of corneal neovascularization following subconjunctival injection in a rat model. However, no significant decreases in inflammation intensity and neovascularization intensity were detected. Furthermore, fibroblast intensity was significantly lower, and there were no significant differences in corneal thickness compared to the control group [235].

In 2008, Ju et al. reported a significantly greater reduction in mouse corneal neovascularization when pegaptanib and PDT were administered simultaneously compared to each monotherapy. This reduction was due to the combined effect of immediate vessel regression caused by PDT and the antiangiogenic effect of pegaptanib. Interestingly, when pegaptanib was given before PDT, the enhanced effect observed with simultaneous treatment was lost. The authors suggested that pegaptanib might interfere with the inflammatory component of PDT, or that the local injection of the aptamer might affect the delivery of verteporfin to the sites of neovascularization, thus reducing its effective concentration. Furthermore, the co-treatment of both agents given simultaneously also resulted in an enhanced reduction in CNV lesion size compared to each treatment alone [236]. Later in 2009, a separate study was conducted to explore the potential synergistic effects of combining irradiation with anti-VEGF treatment on tumor invasion in an orthotopic mouse model of glioblastoma multiforme (GBM). The study utilized the guide screw method for intracranial xenograft of GBM cell lines. Pegaptanib was administered in the tumor bed with or without subsequent irradiation treatment using implanted I-125 seeds. According to the results, locally delivered pegaptanib reduced tumor blood vessel density and increased hypoxia. However, it was ineffective in preventing tumor invasion in the brain. When combined with irradiation of the tumor and the surrounding brain, tumor growth was delayed, and the formation of tumor satellites was suppressed [237].

Pegaptanib’s cellular effects and dose-dependent properties were evaluated on fibroblast-like cells as a model for cellular matrix CNV in vitro. The results showed that pegaptanib had significant antiproliferative activity (survival, proliferative and mitotic activity, and apoptosis) over a 5-day study period. The cellular kinetics demonstrated noticeable dose-dependent effects, with an increase in antiproliferative action as the dose increased [238]. In a study conducted by Carneiro et al., the exact roles of three anti-VEGF agents at clinically used concentrations on distinct steps of angiogenesis in HUVEC were determined. The study found that incubation with pegaptanib did not significantly affect cell proliferation or apoptosis, and only slight decreases were observed in cell migration and the formation of cord structures. However, pegaptanib does play a role in preventing VEGF signaling, as evidenced by a significant reduction in the active form of VEGFR2 (P-VEGFR2) expression and in secreted VEGF [239].

The effectiveness of pegaptanib in the prevention of CNV membrane (CNVM) development was assessed in an experimental rat model. Clinically corresponding doses of pegaptanib were administrated after inducing CNVM through focal laser treatment. The results showed that pegaptanib was ineffective in preventing new CNVM formation, as it only led to a slight reduction in mean CNVM diameters and visual leak-age. Additionally, the pegaptanib groups exhibited greater CNVM mean thickness and clear neovessels infiltrating the retina [240]. Lu and Adelman found that intravitreal pegaptanib sodium does not prevent the leakage associated with laser-induced CNV in rats. The researchers suggested that these results may be attributed to the potential species- and site-specific nature of the response to anti-VEGF therapy. They concluded that the effect of VEGF inhibition can vary in different neovascular animal models [241].

The effects of blocking VEGF on reducing spinal epidural fibrosis were investigated in Wister rats. Three levels of laminectomy were carried out, and the treatment was applied topically to the dura in the surgical field for 5 min using a cotton ball soaked with 3 mg/kg pegaptanib sodium diluted with 0.9% NaCl (1: 10) (treatment group, n = 10). A control group (n = 10) underwent only laminectomy. The results showed that compared to the control group, pegaptanib led to thinner fibrotic tissue on the dura and a significant reduction in spinal epidural fibrosis [242].

Increased levels of VEGF are associated with intervertebral disk (IVD) pathology. VEGF triggers inflammation, contributing to the onset and persistence of pain. Therefore, reducing VEGF levels may help alleviate IVD-related symptoms. In a study conducted by Sato et al., the effects of pegaptanib on pain were explored indirectly by measuring the expression of CGRP in the dorsal root ganglia (DRGs) in a rat model of IVD. The group that received pegaptanib via intervertebral disk puncture showed significantly lower levels of CGRP-positive cells compared to the control-puncture group. In contrast, the control-puncture group exhibited higher CGRP levels compared to the sham group (which did not undergo disk puncture). The authors suggested a VEGFR-1-mediated mechanism of action. Intervertebral disk puncture can cause local inflammation, leading to increased VEGFR levels and inducing CGRP expression. Pegaptanib targets VEGFR-1 to suppress inflammatory cell activation, preventing re-inflammation and suppressing CGRP in the sensory nerve [243].

Dorrell et al. established a link between neural cell death and neovascularization-induced oxidative stress in the retinas of Vldlr −/− mice (a retinal phenotype with dysfunctional receptors for VLDL, which closely corresponds to the vascular features in human diseases associated with photoreceptor cell death). In this study, Pegaptanib reduced intra- and subretinal neovascularization in the deep retina. Moreover, angiostatic combinations were investigated to assess the involvement of abnormal neovascularization in neurodegeneration. Despite the significant reduction in subretinal neovascularization formation, this effect was transient, as subretinal neovascularization levels returned to those of normal Vldlr −/− mice within 2–3 weeks of treatment. The study underscores the necessity of initiating therapy before neovascularization and suggests limitations in the efficacy of antiangiogenic therapy [244].

Klettner and Roider used freshly prepared organ sheets of porcine retina, retinal pigment epithelium, and choroid organ culture, which were cultivated in a perfusion chamber, to directly evaluate the effectiveness of anti-VEGF agents in neutralizing VEGF. The organ sheets were treated with clinically relevant concentrations of pegaptanib (0.08 mg/mL), and the VEGF content of the supernatant was analyzed using ELISA. Interestingly, pegaptanib showed no effect in this system. The authors attributed this outcome to the specific characteristics of both pegaptanib and the study’s design. On one hand, pegaptanib binds to a different site of VEGF that does not interfere with ELISA binding, and therefore, the neutralization could not be seen. On the other hand, due to its binding to the heparin-binding site, pegaptanib does not effectively inhibit the binding of VEGF165 to its receptor site, thereby inhibiting the amplification of VEGF signaling and not VEGFR signaling itself [245].

Mitchell et al. revealed that pegaptanib effectively reduced neovascularization when administered before the induction of the RGS5 gene, a biomarker of pericytes that acts as an indicator of vessel maturation. This anti-VEFG-induced vessel regression was diminished after the induction of the RGS5 gene [246].

A comprehensive study conducted by Foy et al. extensively examined the ocular and systemic safety of pegaptanib sodium in rabbits and dogs. In this study, The animals received intravitreal injections of pegaptanib at dosages that exceeded the levels associated with the recommended dosing regimen for AMD patients (0.3 mg per study eye every 6 weeks). Over nine months, dogs were given doses of 0.3, 1.0, or 3.0 mg every other week, while rabbits received doses of 0.2, 0.67, or 2.0 mg every other week for six months. Throughout the study, no clinical ocular or systemic organ toxicity was observed. Furthermore, the researchers investigated the impact on the cardiovascular system following repeated IV bolus doses in dogs and found no indication of systemic or cardiac complications, further affirming the safe use of pegaptanib for treating ocular neovascular diseases [247]. Another study assessed the safety of varying doses of intravitreal pegaptanib sodium regarding apoptosis in rabbits. The study looked at doses of 0.15, 0.3, and 0.6 mg (the clinical dose and 2–4 times that dose). The effects were measured 14 days and 90 days after a single injection, as well as after repeated monthly injections at the clinical dose for 90 days (0.15 mg). The aptamer was well-tolerated and no changes in the retina were observed by fundus examination and H&E staining. However, a significant increase of 49% in apoptosis was evident 14 days after a single injection of the highest dose. Similar high apoptotic activity was also observed 90 days after repeated injections of the clinical dose (44% after multiple doses of 0.15 mg), indicating that these adverse effects are not dose-dependent [248].

In another study involving rabbits, researchers observed significant changes in normal retina morphology and function after the administration of intravitreal VEGF inhibitors at doses approximately three times higher than those typically used in clinical practice. The study reported an impaired rod-mediated retinal function which was indicated by the significant reduction in b-wave rod-mediated response to dim light 8 weeks after injection compared to the balanced saline solution (BSS) group. This was supported by the absence of significant changes in the amplitude and implicit time of a-wave in response to single-flash white light (representing the photoreceptor function), and further by the morphological findings which showed a normal rhodopsin labeling and a significant reduction in PKC labeling of rod bipolar cells detected 9 weeks postinjection of pegaptanib [249].

Thaler et al. studied the impact of the intravitreal commercially available anti-VEGF therapeutics on the survival of retinal ganglion cells (RGCs) in both healthy and pre-damaged RGC rat eyes. Two different concentrations were used: 10-fold and 60-fold of the clinical doses, respectively, with their respective vehicles serving as controls. The study showed no significant differences in RGC counts, both with and without RGC pre-damage, at both tested concentrations compared to their respective controls for up to 2 months after single injections. However, when using the high concentration of pegaptanib as a treatment in healthy eyes and the respective carrier solution as a control, a striking significant reduction in RGC was observed in both groups after 7 days in comparison to the PBS group. The authors suggested that this outcome could be due to the increased concentrations, which may have changed the chemical composition of the buffer solution, an adverse effect caused by the concentrated solvent, or even the potential systemic absorption of pegaptanib, resulting in an effect on the other eye. In concordance with RGC counts, electron microscopy performed on healthy eyes 2 months after intravitreal injection of the normal concentration of pegaptanib resulted in a significant increase in swelling, disruption of mitochondria, and structural changes indicating RGC damage [250].

Exploring the safety of pegaptanib sodium on isolated bovine retinas showed no short-term toxic effects. The study used electroretinogram (ERG) parameters and found no significant changes in b- or a-wave amplitudes during and after a single exposure to pegaptanib (at 0.006, 0.060, or 0.20 mg/mL) or the solvent carrier alone, indicating its safe application [251].

Christoforidis et al. examined the impact of anti-VEGF therapy on blood-vessel formation during peripheral cutaneous wound healing in a rabbit model. In this study, pegaptanib exhibited minimal inhibition. After 2 weeks, there was a substantial decrease in its efficacy, possibly due to the transition between the proliferative and re-modeling phases of wound healing [252].

The levels of VEGF in the aqueous humor of 16 patients with CNV secondary to AMD were measured before and after intravitreal injections of pegaptanib. An enzyme-linked immunosorbent assay detecting human VEGF121 and VEGF165 was employed. A significant increase in VEGF levels was detected after treatment with pegaptanib. This increase in VEGF levels can be attributed to increased levels of VEGF121, which caused an elevation of the total amount of VEGF measured. However, the researchers observed no correlation between VEGF levels and visual results as VA was enhanced and CFT was decreased after 6 weeks of pegaptanib injection [253].

Manresa et al. evaluated the clinical cardiovascular risk associated with VEGF-inhibiting therapies in 73 patients with exudative AMD, aged 55–81 years, and with no history of anti-VEGF treatment. The evaluation was conducted after 6 weeks using several cardiovascular risk predictors, including homocysteine (Hcy), serum lipids, C-reactive protein (CRP), and fibrinogen levels. In this study, the clinical dose of pegaptanib resulted in no significant increase in cardiovascular risk predictors up to 6 months. However, a significant increase in CRP (in three patients with initially high levels) and a slight, but not significant, increase in fibrinogen levels were observed, which may suggest that anti-VEGF contributes to an alteration of endothelial homeostasis in exudative AMD [254].

A randomized controlled study was conducted to examine the plasma VEGF levels in 30 patients with DME and 30 patients with exudative ARMD before and up to one month after receiving a single intravitreal injection of bevacizumab, ranibizumab, or pegaptanib. In both DME and exudative ARMD patients, there was a significant systematic decrease in plasma VEGF concentrations after 7 days of treatment with bevacizumab, clearly indicating its penetration through all layers of the retina and entry into the systematic circulation This reduction persisted for 4 weeks after the injection with no significant differences in the reduction ratio. However, intravitreal ranibizumab or pegaptanib did not cause any significant changes in plasma VEGF concentrations [255].

In their 2016 study, Takahashi et al. drew attention to the possible inaccuracy of measuring VEGF levels when assessing the effectiveness of anti-VEGF treatments using ELISA kits. The study compared the results of two commonly used ELISA assays with known concentrations of VEGF, in the presence of therapeutic concentrations of four anti-VEGF agents. VEGF-targeting antibodies, not pegaptanib, significantly decreased the measured VEGF concentration compared to the known concentration. Given the mechanism of action of the drugs tested, the researchers proposed a possible competition between those antibodies and the ELISA antibodies at the receptor-binding site [256].

Another study investigated the plasma and vitreous Hcy levels as a leading biomarker for cardiovascular risk. Out of 73 AMD patients included in this study, 37 patients received pegaptanib. Two different control groups were used: 80 healthy subjects (66–80 years old) for comparing plasma Hcy levels, and 40 patients with idiopathic epiretinal membrane for vitreous Hcy levels. Significant differences in the plasma and vitreous Hcy concentrations between AMD patients and the control groups were recorded. After 6 weeks of treatment, no significant changes in the Hcy levels before and after treatment were observed [257].

A population Pharmacokinetics (PK) analysis of intravitreal pegaptanib was conducted in 262 patients with neovascular AMD from studies A5751000, A575100, A5751006, and A5751010 using the 1-compartment model. Predicted parameters from this model indicated that pegaptanib does not accumulate in the plasma after repeated administration. Race, gender, and accompanying diseases such as diabetes, hypertension, and glaucoma, were not determined as influential covariates on pegaptanib PK. However, the analysis did show that creatinine clearance (CLCR) and weight (WT) had a significant impact on pegaptanib clearance (CL) and area under the curve (AUC). Despite this, the changes were deemed not clinically relevant, as a 2.3-fold change in CL over the observed range of CLCR following the administration of a therapeutic 0.3 mg dose of pegaptanib leads to exposures that are one-tenth of those observed with the well-tolerated 3.0 mg dose. Consequently, the study concluded that dose adjustment should not be necessary for patients with moderate renal insufficiency (CLCR > 30 mL/min) and WT > 39 kg [258]. Similar results were found in patients treated for DME as those observed in AMD patients. The empirical or predicted exposure in patients with renal insufficiency (CRCL 30 mL/min) or renal failure (CRCL < 15 mL/min), respectively, indicates no need to adjust the dose regimen (dose and frequency) of pegaptanib [259].

A recent systematic study by Chen et al. unveiled a significant correlation between the expression of NRP1 and the levels of infiltrating immune cells in patients with tumors. This correlation suggests that NRP1 expression may exacerbate the cytokine response, leading to severe symptoms following SARS-CoV-2 infection. Given that NRP1 serves as a crucial factor for SARS-CoV-2 infection and facilitates the virus’s entry into cells, the researchers have advocated for the potential use of NRP1 inhibitors, such as pegaptanib, as therapeutic agents in specific types of cancer, particularly lung and genitourinary cancers, in patients who have experienced COVID-19 [260].

##### Strategies for Maximizing the Potential of Pegaptanib

In a prospective interventional case series report, Lopez-Guajardo et al. proposed an oblique injection technique as a promising alternative to the standard straight intravitreal injection which often leads to reflux and consequent subconjunctival bleb formation, causing a significant loss of the drug administrated and increasing the risk of ophthalmitis. 47 patients were randomized into two groups, the direct injection group (22 eyes) and the oblique injection group (25 eyes). Ultrasound biomicroscopy was used to evaluate the rate of subconjunctival bleb formation 30 min after intravitreal drug delivery. The results showed that the oblique injection group had significantly less subconjunctival bleb formation than the standard technique group. Additionally, there were no significant differences in the rate of injection-related complications between the two groups in this small series [261]. Later, an interventional prospective clinical trial examined the impact of the injection site on vitreal reflux following straight scleral injection. The study included 180 patients with various retinal conditions. The reflux was assessed based on the mean of the width of the broadest conjunctival elevation of the subconjunctival bleb 30 min after injection of triamcinolone acetonide, bevacizumab, or pegaptanib via the superotemporal versus inferotemporal quadrant (6 groups, 30 eyes each). The study concluded that the inferotemporal quadrant resulted in significantly less vitreal efflux than the superotemporal quadrant, with no significant differences observed between the drugs used. Additionally, during the 24-week follow-up period, no significant complications due to intravitreal injection were detected [262].

In 2012, Park et al. developed a method to label the 5′ ends of aptamers with cotinine. This labeling allows the formation of stable complexes with anti-cotinine antibodies, providing an additional affinity unit for biological assays. The researchers used pegaptanib as a model aptamer and successfully used cotinine-conjugated pegaptanib/anti-cotinine antibody complexes in enzyme immunoassays [263]. In 2016, the same research group generated the abovementioned complex (referred to as oligobody) as a novel delivery system to overcome the anti-tumor therapeutic limitations of aptamers. The study found that the inclusion of the antibody led to prolonged in vivo pharmacokinetics of the aptamer without affecting its VEGF-binding affinity. Additionally, the cotinine-aptamer conjugate was released from the oligobody at the target site, enabling deep penetration of pegaptanib into the tumor tissues. Furthermore, the systemic administration of this oligobody in a xenograft mouse model reduced tumor growth to a degree comparable to that of bevacizumab. The proposed mechanism involves the inhibition of tumor angiogenesis and the increase in cancer cell apoptosis. Notably, this antitumor effect was not accompanied by weight loss, indirectly indicating the nontoxic nature of the oligobody, nor were there significant differences in the levels of inflammatory cytokines before and after administration. Furthermore, the observed extended half-life of the cotinine-pegaptanib alone, administered several days after the injection of the anti-cotinine antibody, provided evidence of the formation of this complex in vivo, suggesting a simple and cost-effective aptamer-delivering strategy against a variety of diseases [264].

In 2019, pegaptanib-loaded tetrahedral DNA nanostructure (pegaptanib-TDNs) was introduced by Xie et al. as a novel approach to enhance the anti-angiogenesis and anticancer activity of pegaptanib by improving its cell membrane binding affinity and serum stability. When used at a concentration of 375 nM, pegaptanib-TDNs demonstrated significant antitumor activity by inhibiting the proliferation of HUVECs and Cal27 cells in a concentration-dependent manner. This effect was not detected when pegaptanib was used alone. Additionally, pegaptanib-linked TNDs significantly boosted pegaptanib’s capacity to inhibit the proliferation, migration, and tube formation of VEGF-induced HUVECs [265]. Conventional and advanced methods for administering pegaptanib in the treatment of AMD and cancer are summarized in Table 6.

##### VEGF Isoforms-, Cell-, and Site-Specific Effects of Pegaptanib

In a 2011 study, the proliferation characteristics of isolated human choroidal endothelial cells (hCEC) and retinal endothelial cells (hREC) were assessed following exposure to VEGF isoforms 165 and 121. The research findings indicated that both VEGF isoforms exhibited equivalent potency in stimulating endothelial cell proliferation, with hREC demonstrating a notably higher proliferation rate when subjected to either VEGF isoform than hCEC. This distinction was linked to significant variances in the expression of cell markers. Pegaptanib was observed to effectively counteract the proliferation of hCEC stimulated by VEGF-165. However, it exhibited no effect against VEGF 121-induced proliferation as it is expected [267].

Another study was conducted to assess the effects of VEGF121 and VEGF165, as well as anti-VEGF therapy on RPE permeability. The evaluation was performed on ARPE19 cells for ions, using transepithelial electrical resistance (TEER), and for non-ionic molecules, using fluorescein dextran (FD) permeation assay. The study detected a significant increase in ion permeability (decreased TEER) only after VEGF165 was applied on the apical side, which was counteracted by pegaptanib. However, both VEGF isoforms significantly increased the permeability to 4 kDa dextran, and unexpectedly, pegaptanib induced a statistically significant increase in RPE dextran permeability when administered (pegaptanib and VEGF165, and pegaptanib and VEGF121). The author suggested that these results might be linked to potential changes in the pH and osmolarity of the medium resulting from the drug formulation. Additionally, they highlighted a limitation of the study, noting that it utilized ARPE19, a cell line known to have relatively permeable tight junctions [268].

In a study by Lipp et al., the researchers exploited the selective anti-VEGF-165 isoform (and its murine homolog VEGF 164) effect of pegaptanib to examine the specific roles of different VEGF-A isoforms on hem- and lymphangiogenesis in a murine model of corneal neovascularization. Applying pegaptanib, both topically and systematically, significantly reduced inflammatory corneal hemangiogenesis but not lymphangiogenesis. This indicates the crucial role of VEGF-A 165 for only hemangiogenesis, making pegaptanib a valuable tool for studying the role of lymphatic vessels in various diseases [269].

Magnussen et al. found that VEGF-A165b inhibits angiogenesis and retinal endothelial migration in a dose-dependent manner when induced by 1 nM VEGF-A165 in a mouse model of retinal neovascularization (oxygen-induced retinopathy—OIR). The study also revealed that VEGF-A165b dose-dependently inhibits neovascularization without affecting revascularization. Furthermore, similar to its sister VEGF-A165, VEGF-A165b demonstrated cytoprotective properties for endothelial and epithelial cells both in vivo and in vitro. It is important to note that unlike VEGF-antibodies, which inhibit both VEGF-A165 and VEGF-A165b, pegaptanib selectively binds to the angiogenic form. However, no additional effects were observed from combining pegaptanib and VEGF-A165b [270]. Another study indicated that VEGF-B has a protective effect on the retina, which may help counteract retinal degeneration in various ocular diseases The results showed that VEGF-B significantly inhibited retinal cell death induced by diabetes or intravitreal pegaptanib in rats [271].

##### Higher Order Structure of Pegaptanib

**Figure 6:**
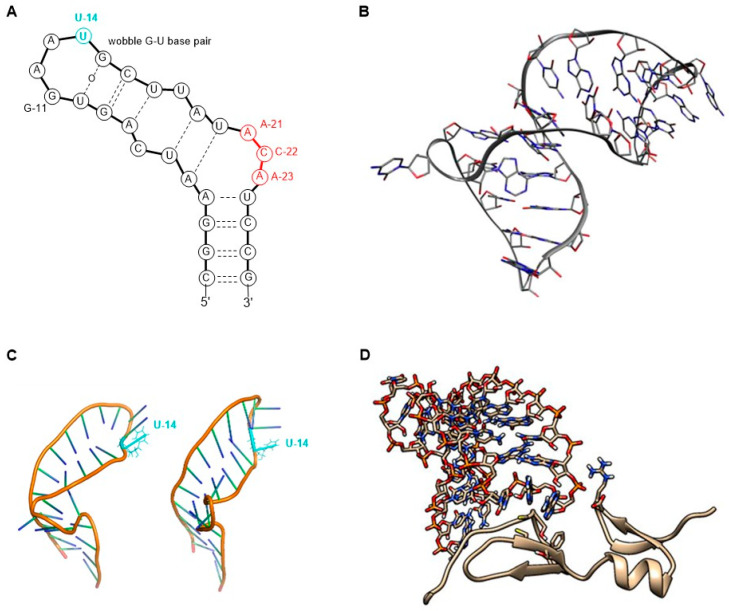
RNA123-predicted secondary (**A**) and tertiary (**B**) structures of the RNA segment of pegaptanib [272], and ToGo WF candidate 3D structures of pegaptanib where U14 is colored in cyan (**C**), and the predicted structure of the pegaptanib–VEGF complex after rebuilding the full-length RNA (**D**) [273].

Efforts have also been made to uncover the tertiary structure of pegaptanib. In 2014, Eriksson et al. employed RNA123 program to predict the tertiary structure of the pure nucleic acid sequence of the aptamer. After validating the accuracy of the de novo prediction of this program, the four most optimal secondary structures along with their corresponding tertiary structures were generated. Figure 6 illustrates the structure with the highest similarity to the one indicated by NMR experiments. The predicted structure displays a helix structure with the four canonical base pairs, a bulge of the non-interacting bases A-21–C-22–A-23, and a G-11–U-14 hairpin tetraloop structure with two canonical and one wobble G–U base pairs prior to the hairpin loop. U-14 is situated in the hairpin loop region, which points towards a considerable pocket formed by a significant fold in the helix. This pocket is likely where VEGF tightly binds, enabling the interaction of U-14 with VEGF [272].

In 2019, Yamasaki et al. developed a workflow that integrated various RNA-related analytical methods to predict the tertiary structure of the RNA segment of pegaptanib and its interaction with VEGF. They identified two potential structures with open internal-loop formations for A-4–C-7 and U-18–U-24, and with U-14 base facing outward. Subsequently, docking calculations of the pegaptanib fragment A-13–U-14–G-15 and VEGF were performed, and the illustrated structure showed the closest distance between the centroid of base heavy atoms of U-14 and the centroid of the Cys10-Cys27 disulfide bond of VEGF, which was consistent with the experimental results. This structure was then used for simulations of substitution mutations where U14 was replaced by A, G or C to investigate the stability and affinity of the complex. According to the results, U-14 showed the shortest distance, while U-14G had the highest binding affinity, providing new insight into the design of aptamers with enhanced affinity [273].

## 3. Aptamers Beyond Pegaptanib—The Latest Breakthrough in AMD and Emerging New Applications

Nearly 20 years since the landmark approval of the first aptamer, the FDA granted its approval to the second aptamer, avacincaptad pegol (Izervay TM), on 4 August 2023. Avacincaptad pegol is a pegylated 39mer RNA aptamer developed to combat geographic atrophy (GA) secondary to AMD, heralding a new era of treatment options for this condition and sparking renewed interest in aptamers within the industry [274,275]. The recently approved aptamer targets the complement system by blocking the cleavage of the C5 complement protein into C5a and C5b. Primarily, this inhibition prevents the assembly of the membrane attack complex (MAC, C5b-9), which causes cell lysis and death [275,276]. This approval is supported by the positive outcomes from two Phase III clinical trials, GATHER1 and GATHER2. These trials assessed the safety and effectiveness of a monthly 2 mg intravitreal treatment versus sham control in patients with GA secondary to AMD. The treatment led to a reduction in the growth rate of GA by up to 35% after 12 months. Avacincaptad pegol is well-tolerated, and some observed adverse effects include conjunctival hemorrhage, increased IOP, and blurred vision [275,276].

Platelet-derived growth factor (PDGF) plays an essential role in angiogenesis [277], and inhibition of PDGF results in a loss of pericytes, making endothelial cells more susceptible to anti-VEGF treatment [278]. Therefore, simultaneous blocking of VEGF-A and PDGF can be more effective in inhibiting the formation of neovascularization [48]. E10030 (Fovista^®^) is a pegylated DNA-aptamer that targets PDGF and has been used in combination with ranibizumab for treating AMD. Phase I and II clinical trials have shown that combining E10030 with ranibizumab is more effective than using ranibizumab alone. These positive outcomes allowed further evaluation of its safety and efficacy in Phase III studies in combination with various anti-VEGF therapies [43,48].

A Few other aptamers are currently under clinical investigation for their potential use in treating AMD. These include the RNA aptamer umedaptanib pegol (APT-F2P, RBM-007), which targets fibroblast growth factor 2 (FGF2) [279,280,281], as well as the DNA aptamer AS1411, which binds to cell surface nucleolin [282].

In addition to AMD treatment, aptamers are considered promising therapeutic agents in various fields, including the treatment of cancer, neurodegenerative diseases, immune system disorders and inflammation, as well as viral and bacterial infections [283,284,285]. Moreover, aptamers can be used to deliver various therapeutic reagents to target cells and tissues, due to their high target specificity and affinity [285,286]. Aptamer-mediated delivery systems are currently being developed for cancer treatment including aptamer-drug conjugates, aptamer-siRNA conjugates, and aptamer-functionalized nanomaterials [287]. A list of therapeutic aptamers currently undergoing clinical development is presented in Table 7.

Aptamers can be delivered through various routes, including intravitreal, intravenous, subcutaneous, and oral administration. Among these methods, the intravenous route is the most commonly employed for the delivery of aptamers (Table 7). However, this approach has certain drawbacks, such as the rapid clearance from the circulatory system [3]. Recent research has underscored the potential application of aptamers in combating SARS-CoV-2 infections [288]. Notably, the ssDNA aptamer BC-007 has advanced to phase II clinical trials for the treatment of long COVID-19. The intranasal administration of aptamers offers a non-invasive delivery option, and the formulation of aptamers into gels and aerosols represents a strategic approach for the targeted delivery of these molecules to the respiratory tract [289]. Oral administration is an appealing non-invasive route; however, it presents significant challenges due to the gastrointestinal barrier, which can impede the therapeutic efficacy of aptamers as a result of their limited bioavailability [290].

Advancements in biomaterials and nanotechnology have substantially boosted the research of biodegradable microparticles and nanoparticles designed to encapsulate aptamer-based therapeutics. Such delivery systems enhance cellular uptake, increase stability within biological environments, and facilitate intracellular targeting [291]. While intravenous injection is typically considered the preferred method for administering aptamer-conjugated nanocarriers, many studies are also exploring the potential of topical administration for the delivery of aptamer-functionalized carriers in the treatment of skin cancer [292].

## 4. Discussion

The development of pegaptanib has been a landmark achievement in the history of nucleic acid therapeutics, affirming the role of aptamers as versatile biological agents. Furthermore, pegaptanib has had a significant impact on the development of aptamers. The chemical modifications used in pegaptanib are widely applied in other aptamers, such as avacincaptad pegol and umedaptanib pegol. These structural alterations include 5′-PEGylation via an aminohexyl linker, 2′-deoxo-2′fluoro nucleosides, 2′-OMe nucleosides, and 3′-capping with inverted deoxythymidine.

As many proteins are expressed in various isoforms, specific pathological isoforms will be identified in other diseases. The remarkable specificity of aptamers positions them as powerful tools for effectively distinguishing between these isoforms, paving the way for more targeted therapeutic approaches.

The history of pegaptanib is somewhat similar to that of fomivirsen [293]. Both were pioneers in multiple areas: fomivirsen was the first approved gene-silencing medicine, the first approved antisense oligonucleotide, and the first approved DNA-based therapeutic. Meanwhile, pegaptanib was the first approved aptamer, the first anti-VEGF agent, and the first RNA-based medicine. Both were demonstrated to be effective and safe but were eventually crowded out of the market by more favored competitors. However, there are significant differences between them. Fomivirsen was from the outset a second-line treatment for a rare disease, while pegaptanib was at one point the best available therapeutic option for a relatively common condition. The fall of fomivirsen did not hinder the advancement of gene-silencing technology across various indications [35,293]. In contrast, almost 20 years passed after pegaptanib’s approval in 2004 before a second aptamer was approved for a similar condition [274,275]. From the rival group of antibodies, several new molecules were authorized, including brolucizumab [294].

The encouraging preliminary results derived from both animal studies and clinical trials prompted the FDA to grant fast-track status to pegaptanib for the treatment of AMD. The Phase III clinical trials revealed modest effects, particularly in stabilizing patients’ vision and reducing the subretinal fluid. This compelling evidence ultimately led to the FDA approval of pegaptanib.

Despite the promising potential of aptamers as therapeutic agents, only a limited number of candidates have reached clinical trials (Table 7). The relatively quiet years in aptamer research can be attributed to several factors including the exclusive early intellectual property rights for SELEX technology and the high costs and intensive labor associated with suboptimal SELEX procedures. Furthermore, researchers tended to concentrate on existing aptamers, such as the thrombin aptamer, to investigate novel design strategies rather than dedicating resources to the isolation of new aptamers [295,296]. This shift in focus has further hindered the discovery of new aptamers that could expand the available tools for scientific and medical applications.

Optimizing the selection process presents a significant challenge that hinders the advancement of aptamers in clinical settings [297]. Recent developments in high-throughput sequencing techniques have enabled the screening of a wide range of nucleic acid aptamer candidates using High-Throughput SELEX (HT-SELEX) approaches. However, given the expansive potential sequence space, identifying aptamers that selectively bind to target proteins continues to be a challenging task [298,299]. In silico approaches, particularly molecular modeling and molecular dynamics, have recently emerged as viable alternatives to SELEX. The integration of these computational techniques with HT-SELEX significantly enhances the effectiveness of research efforts. Using these methods, researchers can design aptamers targeting simple compounds and macromolecules. Nevertheless, a notable limitation resides in the current state of molecular modeling, which does not allow the use of cells as targets in aptamer design—a distinct advantage offered by the in vitro SELEX method. Through molecular modeling techniques, it is feasible to identify new aptamers with improved affinity for specific targets and define aptamer–target interactions. This feature is particularly advantageous, given that point mutations can further improve the affinity of the aptamer [299].

The therapeutic use of aptamers is limited not only by their specificity and affinity for the target but also by their poor pharmacokinetics, such as instability in biological environments, rapid clearance from circulation, and insufficient tissue penetration. These issues often necessitate frequent injections, which can result in various side effects. Incorporating aptamers within innovative drug delivery systems such as liposomes, nanoparticles, and polymeric micelles can help protect aptamers from enzymatic cleavage and clearance, thereby extending their half-life and improving their bioavailability. Additionally, these delivery systems facilitate the controlled release of therapeutics, effectively reducing the frequency of administration. By employing surface modifications with targeting ligands or antibodies, these systems enable targeted delivery to the affected cells, thereby minimizing the side effects and improving the therapeutic potential [291].

Aptamer technology has evolved extensively since the development of pegaptanib in the late 1990s. The approval of avacincaptad pegol, along with the advancement of other aptamers in clinical phases, indicates that despite the early challenges faced by the first aptamer, there is still considerable potential in this area, and we may be on the cusp of a new renaissance for aptamers [300].

## Figures and Tables

**Table 1:** Comparing aptamers and antibodies.

Properties	Aptamers	Antibodies
chemical structure	oligonucleotide (RNA or DNA)	protein
size	6–30 kDa	150–180 kDa
target	wide range of molecules ^a^	wide range of molecules ^b^
specificity	high	high
affinity	high	high
chemical stability	higher	lower
thermal stability	stable up to 80 °C ^c^	Low ^d^
stability in biological systems	Low	higher
batch-to-batch variation	lower	higher
shelf life	long	shorter
immunogenicity	low, even in excess of therapeutic doses ^e^	higher, especially in high doses ^f^
synthesis	chemical (SPOS or enzymatic) and, to a lesser extent, biological	biological
development and synthesis cost	cheaper	more extensive
usage of modified building blocks	possible	not possible
after synthesis modifications	easier	more difficult

Green indicates the advantage, while red indicates the disadvantage of the group (aptamer or antibody) over the other in the given aspect. SPOS: solid-phase oligonucleotide synthesis. ^a^ Negatively charged molecules are not favored, ^b^ except for toxic or nonimmunogenic molecules, ^c^ reversible denaturation, ^d^ irreversible denaturation, ^e^ except for phosphorothioate-modified molecules, ^f^ except for humanized antibodies.

**Table 2:** Phase I-III efficacy results of pegaptanib.

Phase	Type	Outcome	Dosage	Duration	N	Results	Ref
Ia	multicenter, open-label, dose-escalation, and safety study	3 lines or greater increase in vision on ETDRS chart	single dose varying from 0.25 to 3.0 mg	3 months	15	4/15 patients (26,7%) ^a^	[56]
II	multicenter, open-label repeat dose study	3 lines or greater increase in vision on ETDRS chart	3 mg 3 times at 28-day intervals	3 months	10 + 11 ^b^	25% and 60% ^a,b^	[59]
III	prospective, randomized, double-blind, multicenter, dose-ranging, controlled	losing < 15 letters	0.3, 1, or 3 mg, or sham	54 weeks	1186 (294, 300, 296, 296)	70%, 71%, 65%, 55%	[61]

The different doses of pegaptanib and sham, and their respective data, are color-coded. ^a^: ratio of patients who showed a significant increase in vision according to the indicated outcome, ^b^: results for pegaptanib alone and pegaptanib + photodynamic therapy, respectively.

**Table 3:** Key findings on the efficacy of pegaptanib in AMD.

Type	Outcome	Duration	N	Results	Ref
Multicenter, randomized, double-masked, sham-controlled study	mean change in VA loss > 15 letters progressing to legal blindness	48 weeks ^a^	111 vs. 116	maintained 7% vs. 14% 34%→35% vs. 24%→38%	[68]
Exploratory analysis of the VISION study results	loss < 15 letters	54 weeks	34 and 30 ^b^	76% vs. 50% and 80% vs. 57% compared to sham	[75]
Respective review of data	gain > 3 lines stabilized VA loss > 3 lines	6–14 months	90	20%, 70%, 10%	[76]
Interventional case report	CNV progression	6 weeks	2	CNV progressed in both cases	[77]
Prospective, nonrandomized, observational case series	CFT leakage VA	12 weeks	41	340 ± 24 μm→299 ± 14 μm 100%→81% 20/116→20/120	[79]
Longitudinal observation	mean logMAR VA change	30.5 ± 8 weeks	14	0.67 ± 0.3→0.74 ± 0.16	[80]
Retrospective data analysis	mean VA change	6 month	50	0.37 ± 0.24→0.4 ± 0.26	[81]
Noncontrolled study	loss < 3 lines	24 weeks	27	89%	[82]
Retrospective, interventional, comparative study	mean logMAR VA change foveal thickness	12 months	43	1.25 ± 0.43→0.83 ± 0.44 452.3 ± 44.83 μm→274.3 ± 13.33	[83]
Retrospective review	mean VA change gain > 3 lines loss < 3 lines	21 weeks	73	−0.68 lines 16% 70%	[84]
Retrospective review	median VA change	24 months	5	20/200→20/60	[85]
Retrospective review	loss < 15 letters	54 weeks	62	90%	[86]
Noncontrolled study	mean VA change gain > 3 lines loss > 6 lines	5.2 ± 1.5 months	41	−0.03 lines 17.1% 7.31%	[87]
Noncontrolled study	loss < 15 letters gain > 0 letters gain > 15 letters	52 weeks	56	79% 43% 9%	[89]
Retrospective, interventional case series	mean VA improvement	6 months	22	2.2 lines or 0.7 lines ^c^	[91]
Prospective, open-label, non-comparative, observational case series	mean VA change CNV area change	6 months	7	−5 letters 1.4 mm^2^→2.7 mm^2^	[92]
Randomized comparative	mean VA change	13.93 ± 5.87 months	3 × 15 ^d^	0.3, 1.0, 2.2 ^d^	[93]
Randomized comparative	stable VA	6 months	13: 18: 17 ^e^	61.5%, 55.5%, 76.5% ^e^	[94]
Phase IV, prospective, uncontrolled study	mean VA improvement	54 weeks	568	15.9 letters	[95]
Prospective, uncontrolled study	mean logMAR VA	54 weeks	75	0.26 ± 0.24→0.29 ± 0.28 ^f^	[96]
Prospective, uncontrolled study	mean logMAR VA CFT	3 years	16	0.24 ± 0.25→0.25 ± 0.28 ^f^ 232 ± 54 μm→210 ± 59 μm ^f^	[97]
Retrospective review	loss < 15 letters	10–16 months	80	97%	[98]

Color codes indicate the achieved results for multiple listed outcomes and data from different groups. VA = visual acuity. ^a^: continuation of an earlier study; patients were re-randomized after 54 weeks of treatment for 48 weeks— results in this table are compared to those of discontinued treatment. ^b^: two groups were formed based on two sets of clinical characteristics typical of early disease. ^c^: in the previously non-treated and the previously treated groups, respectively. ^d^: patients in three groups received either PDT + pegaptanib, PDT only, or pegaptanib only; the results are displayed respectively. ^e^: the three groups received bevacizumab, pegaptanib, or bevacizumab followed by pegaptanib, respectively. ^f^: from the start of the maintenance therapy until the end of the follow-up.

**Table 4:** Key findings on the efficacy of pegaptanib in DR and DME.

Type	Outcome	Duration	N	Results	Ref
Phase II, randomized, double-masked, dose-ranging controlled trial	median VA gain > 10 letters mean CRT change	36 weeks	172	20/50 vs. 20/63 ^a^ 34% vs. 10% ^a^ −68 μm vs. +4 μm ^a^	[100]
Retrospective analysis of a randomized clinical trial.	changes in retinal neovascularization	36 weeks	16	62% showed regression in the pegaptanib group and 0 in the sham group	[101]
Retrospective analysis	mean VA change (logMAR)	varying	13 12 15	−0.16 ± 0.15 (pegaptanib) −0.06 ± 0.14 (combined treatment) +0.03 ± 0.13 (photocoagulation)	[103]
Retrospective analysis	mean CRT change	varying	13 12 15	−146.77 ± 93.39 (pegaptanib) −71.67 ± 105 (combined treatment) +19.2 ± 54.2 (photocoagulation)	[103]
Prospective, interventional, single case report	CRT VA	42 months	1	511 μm→376 μm stable	[104]
Case report	VA	15 months	1	30/200→180/200	[105]
Retrospective analysis	mean VA mean CRT	6–12 months	63	0.66 ± 0.37→0.53 ± 0.37 533.1 ± 123.1→373.7 ± 135.8	[106]
Prospective, randomized, controlled, exploratory study	NV regression VA change	30 weeks	20	complete regression in all eyes vs. in 20% of eyes +5.8 letters vs. −6.0 letters	[107]
Phase II/III, sham-controlled trial	gained letters	2 year	207	6.1 vs. 1.3	[109]
Randomized controlled trial	gain > 10 letters	54 weeks	260	36.8% vs. 19.7%	[110]
Prospective, interventional study	FAZ size change	36 weeks	30	no significant change	[111]
Longitudinal, interventional, non-randomized study	mean reduction in CFT VA	48 weeks	30	551.5 ± 129.8→237.4 ± 41.1 18.2 ± 8.5 →25.5 ± 8.4 letters	[112]
Prospective case series	VA change	18 weeks	8	no significant change	[113]

Color codes indicate the achieved results for multiple listed outcomes and data from different groups. VA = visual acuity, CRT = central retinal thickness, NV = neovascularization, FAZ = foveal avascular zone, ^a^: the results of the group receiving 0.3 mg pegaptanib vs. the sham group are displayed.

**Table 5:** Key findings of clinical trials assessing the efficacy of pegaptanib in comparison to anti-VEGF antibodies.

Treatment	Outcome	Duration	N	Results	Ref
Pegaptanib (0.3 mg) vs. sham	loss < 15 letters	54 weeks	1186	70% vs. 55%	[61]
Ranibizumab (0.3 mg) Ranibizumab (0.5 mg) PDT	loss < 15 letters	12 months	423	94.3% 96.4% 64.3%	[208]
Ranibizumab (0.3 mg) ranibizumab (0.5 mg) Verteporfin	loss < 15 letters	24 months	716	90% 92% 52.9%	[209]
Ranibizumab + PDT vs. sham + PDT	loss < 15 letters	12 months	162	90.5% vs. 67.9%	[210]
Ranibizumab + PDT vs. sham + PDT	loss < 15 letters	24 months	162	88% vs. 75%	[211]
Bevacizumab (no control)	mean VA	8 weeks	81	20/200→20/125	[214]
Bevacizumab vs. ST	loss < 15 letters	54 weeks	131	91% vs. 67%	[216]
Bevacizumab vs. pegaptanib	retinal edema reduction	54 weeks	122	−0.82 mm^3^ vs. −0.31 mm^3^	[217]
Bevacizumab vs. pegaptanib	total retinal volume reduction	3 months	53	−0.88 ± 1.4 mm^3^ vs. −0.07 ± 0.5 mm^3^	[218]
Bevacizumab vs. pegaptanib	gained letters FT change	6 months	15	+7.2 vs. +9.1 −52.9 μm vs. −88.2 μm	[219]
Pegaptanib vs. ranibizumab	mean VA change	12 months	81	−0.095→−0.18 vs. −0.077→−0.11	[221]

Color codes indicate the achieved results for multiple doses of ranibizumab and multiple listed outcomes. PDT = photodynamic therapy, ST = standard treatment, FT = foveal thickness, IVTA = intravitreal triamcinolone acetonide, VA = visual acuity.

**Table 6:** Conventional and advanced treatments/delivery systems for administering pegaptanib.

Treatment/Delivery System	Disease	Advantages	Route of Administration	Ref
**Conventional**				
Straight intravitreal injection	AMD	-	Intravitreal injection	
Oblique intravitreal injection		Less vitreal efflux		[261]
Straight scleral injection via the inferotemporal quadrant		Less vitreal efflux		[262]
**Advanced**				
Biodegradable PLGA microspheres + lyophilization of the aptamer in the presence of trehalose before encapsulation	AMD	Sustained release of pegaptanib Co-encapsulation with trehalose maintains aptamer integrity after the release	Intravitreal injection	[58,266]
Cotinine-conjugated pegaptanib/anti-cotinine antibody complexes	Cancer	Overcoming poor pharmacokinetic lifetimes and enhancing therapeutic potential	Intraperitoneal or Intravenous injection	[264]
Pegaptanib-loaded tetrahedral DNA nanostructure (pegaptanib-TDNs)		Enhancing therapeutic potential by improving pegaptanib’s binding affinity and serum stability	-	[265]

PLGA = poly lactic-co-glycolic Acid.

**Table 7:** Aptamers under clinical development.

Aptamer	Disease	Development Stage and Trial ID	Route of Administration
Pegaptanib	Neovascular AMD	FDA-approved	Intravitreal
Avacincaptad pegol	GA secondary to AMD	FDA-approved	Intravitreal
	Autosomal recessive Stargardt disease	Phase IIb [NCT03364153]	
Umedaptanib pegol (APT-F2P, RBM-007)	Neovascular AMD	Phase II [NCT04200248, NCT04640272]	Intravitreal
	Achondroplasia	Phase II [jRCT2031220338]	Subcutaneous
AS1411	Acute Myeloid Leukemia	Phase II [NCT00512083]	Intravenous
	Advanced solid tumors	Phase I [NCT00881244]	
BC-007	Dilated cardiomyopathy	Phase II [NCT04192214]	Intravenous
	Long COVID-19	Phase II [NCT05911009]	
Rondaptivon pegol (BT200)	Von Willebrand Diseases, Hemophilia A	Phase II [NCT04677803]	Subcutaneous or intravenous
	Von-Willebrand disease Type 2B	Phase II [2024-518294-34-01]	
AON-D21	Community-acquired pneumonia	Phase II [NCT05962606]	Intravenous
Olaptesed pegol (NOX-A12)	Glioblastoma	Phase I/I [NCT04121455]	Intravenous
	Colorectal and pancreatic cancer	Phase I/II [NCT03168139]	
	Chronic Lymphocytic Leukemia	Phase II [NCT01486797]	
	Multiple Myeloma	Phase II [NCT01521533]	
NOX-E36	Type 2 Diabetes Mellitus	Phase I/II [NCT01085292]	Subcutaneous
	Type 2 Diabetes Mellitus and Albuminuria	Phase II [NCT01547897]	
Lexaptepid Pegol (NOX-H94)	Anemia, end-stage renal disease	Phase I/II [NCT02079896]	Subcutaneous or intravenous
	Anemia of Chronic Diseases	Phase II [NCT01691040]	
ApTOLL	Acute ischemic stroke	Phase Ib/IIa [NCT04734548]	Intravenous
BB-031	Acute ischemic stroke	Phase II [NCT06226805]	Intravenous
AM003	Solid tumors	Phase I [NCT06258330]	Intratumor route
Apta-1	Sepsis, Inflammation	Phase I [ISRCTN15455814]	Intravenous
